# Bioprospecting of Actinobacterial Diversity and Antibacterial Secondary Metabolites from the Sediments of Four Saline Lakes on the Northern Tibetan Plateau

**DOI:** 10.3390/microorganisms11102475

**Published:** 2023-10-01

**Authors:** Shao-Wei Liu, Xiao-Xu Zhai, Di Liu, Yu-Yu Liu, Li-Ying Sui, Ke-Ke Luo, Qin Yang, Fei-Na Li, Arina A. Nikandrova, Arina N. Imamutdinova, Dmitrii A. Lukianov, Ilya A. Osterman, Petr V. Sergiev, Ben-Yin Zhang, De-Jun Zhang, Chun-Mei Xue, Cheng-Hang Sun

**Affiliations:** 1 Department of Microbial Chemistry, Beijing Key Laboratory of Antimicrobial Agents, Institute of Medicinal Biotechnology, Chinese Academy of Medical Sciences, Peking Union Medical College, Beijing 100050, China; liushaowei3535@163.com (S.-W.L.);; 2 State Key Laboratory of Plateau Ecology and Agriculture, Qinghai University, Xining 810016, China; 3 College of Life Sciences, Jiamusi University, Jiamusi 154000, China; 4 College of Marine and Environmental Sciences, Tianjin University of Science and Technology, Tianjin 300457, China; 5 Laboratory of Respiratory Diseases, Beijing Key Laboratory of Pediatric Respiratory Infection Diseases, Beijing Pediatric Research Institute, Beijing Children’s Hospital, Capital Medical University, Key Laboratory of Major Diseases in Children, Ministry of Education, National Clinical Research Center for Respiratory Diseases, National Center for Children’s Health, Beijing 100045, China; hbulifeina@126.com; 6 Center of Life Sciences, Skolkovo Institute of Science and Technology, 121205 Moscow, Russia; 7 Department of Biology, Lomonosov Moscow State University, 119991 Moscow, Russia; 8 Department of Chemistry, Lomonosov Moscow State University, 119991 Moscow, Russia; 9 College of Eco-Environmental Engineering, Qinghai University, Xining 810016, China; benyinzhang@qhu.edu.cn (B.-Y.Z.); djzhang@qhu.edu.cn (D.-J.Z.)

**Keywords:** saline lakes, actinobacteria, Northern Tibetan Plateau, antibacterial metabolites, MATRIX cultivation, molecular networking

## Abstract

The Tibetan Plateau, known as the “Roof of the World” and “The Third Pole”, harbors numerous saline lakes primarily distributed in the Northern Tibetan Plateau. However, the challenging conditions of high altitude, low oxygen level, and harsh climate have limited investigations into the actinobacteria from these saline lakes. This study focuses on investigating the biodiversity and bioactive secondary metabolites of cultivable actinobacteria isolated from the sediments of four saline lakes on the Northern Tibetan Plateau. A total of 255 actinobacterial strains affiliated with 21 genera in 12 families of 7 orders were recovered by using the pure culture technique and 16S rRNA gene phylogenetic analysis. To facilitate a high-throughput bioactivity evaluation, 192 isolates underwent OSMAC cultivation in a miniaturized 24-well microbioreactor system (MATRIX cultivation). The antibacterial activity of crude extracts was then evaluated in a 96-well plate antibacterial assay. Forty-six strains demonstrated antagonistic effects against at least one tested pathogen, and their underlying antibacterial mechanisms were further investigated through a dual-fluorescent reporter assay (pDualrep2). Two *Streptomyces* strains (378 and 549) that produce compounds triggering DNA damage were prioritized for subsequent chemical investigations. Metabolomics profiling involving HPLC-UV/vis, UPLC-QTOF-MS/MS, and molecular networking identified three types of bioactive metabolites belonging to the aromatic polyketide family, i.e., cosmomycin, kidamycin, and hedamycin. In-depth analysis of the metabolomic data unveiled some potentially novel anthracycline compounds. A genome mining study based on the whole-genome sequences of strains 378 and 549 identified gene clusters potentially responsible for cosmomycin and kidamycin biosynthesis. This work highlights the effectiveness of combining metabolomic and genomic approaches to rapidly identify bioactive chemicals within microbial extracts. The saline lakes on the Northern Tibetan Plateau present prospective sources for discovering novel actinobacteria and biologically active compounds.

## 1. Introduction

Actinobacteria produce the majority of naturally occurring antibiotics, and approximately two-thirds of naturally derived antibiotics in current clinical use were produced by actinobacteria [1,2]. As magic bullets, antibiotics have always been the most effective antimicrobials [3,4]; however, the bottleneck of rediscovery of known strains and antibiotics from the actinobacteria dwelling in traditional environmental niches decreases the efficiency of new antibiotic discovery and pushes researchers to shift the bioprospecting of pharmaceutical actinobacteria to underexplored genera and ecological niches or untapped sources [5,6], such as rare actinobacteria or extreme geographical locations including deserts [7], mangroves [8], caves [9], deep-sea [10], etc. [11]. However, the antibiotic discovery of actinobacteria isolated from saline lakes has thus far been minimal, which needs more research to focus on the source [12,13].

Saline lakes are widely distributed in the world and are categorized into three zones based on their location, including the southern hemisphere lake zone, the northern hemisphere lake zone, and the equatorial lake zone [14]. In addition to being hypersaline, saline lake ecosystems are often characterized by other harsh environmental conditions, such as high alkalinity, low oxygen concentration, low nutrient availability, and high solar irradiation, depending on the geographical area [15,16]. Although considered to be a hostile environment for the growth of most organisms, saline lakes are often teeming with a large diversity of microbial communities, including bacteria, archaea, viruses, and eukaryotes [17,18]. Over the past two decades, extensive studies based on culture-dependent and independent techniques have been conducted to survey the microbial diversity of the saline lake environments, as exemplified by the Great Salt Lake in the United States [19], the Dead Sea [20], Chott El Jerid Lake in Tunisia [21], Meyghan Lake in Iran [22], the Solar Lake in Egypt [23], Chaka Lake, Keke Lake, and Aiding Lake in China [24,25,26]. Moreover, groups of novel halophilic or halotolerant bacteria found in saline lakes have been described. To the best of our knowledge, up to the end of January 2022, a total of 64 novel species of actinobacteria, including two new orders (*Nitriliruptorales* [27] and *Jiangellales* [28,29]), three new families (*Nitriliruptoraceae* [27], *Jiangellaceae* [28,29] and *Ruaniaceae* [30]) and 13 new genera, have been reported from the inland saline environment.

Microorganisms inhabiting inland saline environments have been reported to produce versatile substances of interest, such as hydrolytic enzymes, compatible solutes, pigments, and antibiotics [31]. Some anti-tumor and antimicrobial substances have been discovered from actinobacteria inhabiting saline environments, such as salternamides A-E [32,33], salinazinones A-B [34] and xiamycins C-E [35] from *Streptomyces*; erythronolides H-I [36] and actinopolysporins A-C [37] from *Actinopolyspora*; and borrelidins C-E [38] and nocarbenzoxazoles A-G [39] from *Nocardiopsis*. Chemical types of these entities are composed of anthraquinones, benzofurans, sesquiterpenoids, oxazinones, macrolides, etc. However, reports on the bioactive metabolites produced by actinobacteria from the inland saline environment are still scant, which is incomparable with those reported from other extreme environments such as deserts, mangroves, and deep seas [40]. Therefore, the pharmaceutical potential of actinobacteria residing in pristine saline lakes needs to be further deeply explored.

China is one of the countries with the most extensive distribution of saline lakes. These lakes spread over four regions: the Qinghai-Tibetan Plateau region, the Northwestern region, the North-central region, and the Eastern region [41]. Tibet is situated on the Qinghai-Tibetan Plateau, which is known as the “Roof of the World” and “The Third Pole” due to its average height above sea level of over 4000 m, and it is the second-largest store of ice on Earth after the Arctic or Antarctic [42]. The Northern Tibetan Plateau, also known as “Qiangtang Plateau," is the main part of the Qinghai-Tibetan Plateau, covering about 0.6 million square kilometers with an altitude exceeding 4500 m above sea level [43]. There are approximately 221 saline lakes in Tibet, with the vast majority (214) distributed in the Northern Tibetan Plateau (Qiangtang Plateau) [44]. However, the challenging climate, low oxygen levels, high radiation, and shortage of supplies in this region make it extremely formidable for researchers to explore its scientifically valuable resources, such as saline lakes. Therefore, exploration of the actinobacterial diversity of the saline lakes on the Northern Tibetan Plateau is quite rare, and their associated bioactive secondary metabolites are hitherto uncharacterized.

The present study focuses on the exploration of actinobacterial strains as potential pharmaceutical resources for the production of biomolecules with antimicrobial or anti-tumor properties in extreme or unusual environments instead of traditional and easily accessible environments. Specifically, we isolated cultivable actinobacteria from the sediments of four saline lakes located on the Northern Tibetan Plateau, namely Dangqiong Co., Dong Co., Nieer Co., and a nameless saline lake. To assess the antibacterial activity of these isolates against the “ESKAPE” [45] pathogens, the OSMAC approach in conjunction with a 24-well microbioreactor system, known in-lab as MATRIX cultivation [46,47] was employed. Multi-omics approaches, including UPLC-QTOF-MS/MS-based metabolomics profiling, molecular networking, and genome mining, were utilized to identify potential secondary metabolites produced by two *Streptomyces* strains with DNA-damage activity. The findings of this study could provide valuable information for bioprospecting new sources of antibiotics for further research in the saline lakes of China.

## 2. Materials and Methods

### 2.1. Samples Collection and Preparation

**Figure 1:**
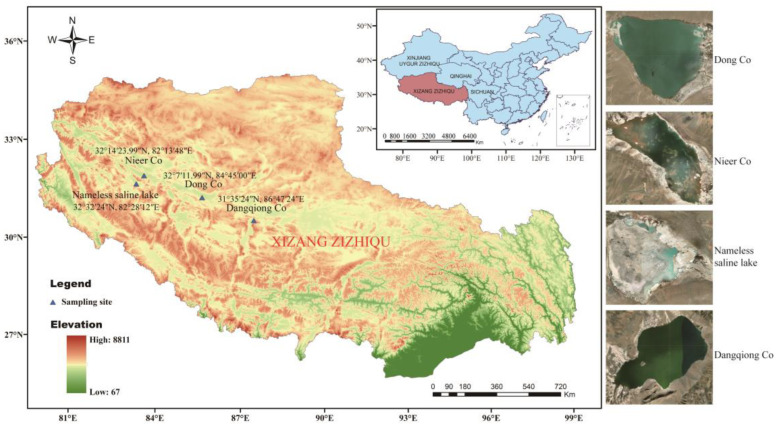
Geographic distribution of the sampling sites from four saline lakes on the Northern Tibetan Plateau in China.

Four sediment samples were separately collected at a depth of 5–15 cm in four saline lakes on the Northern Tibetan Plateau and then loaded into 50 mL sterile Falcon tubes. Samples are kept on ice until transported to the lab for further processing. Samples are air-dried in a laminar flow hood for 8 h at room temperature prior to separation. The geographic location of the four saline lakes and information about sampling sites are shown in Table S1 and Figure 1.

### 2.2. Actinobacteria Isolation

Actinobacteria were isolated by using the serial dilution method and the spread plate technique. Briefly, 1 g of sediment sample was suspended in 10 mL of sterile distilled water, and the solution was mixed in a rotatory shaker at 180 rpm at room temperature for an hour. The mixed suspension was then diluted with sterile distilled water in ten-fold series to the final concentrations of 10^−2^, and 10^−3^ g/mL, and 200 μL of the dilutions (10^−1^, 10^−2^, and 10^−3^ g/mL) from each sediment sample were spread onto eight different isolation media (Table S2). All media were supplemented with nalidixic acid at a final concentration of 20 μg/mL, cycloheximide (50 µg/mL), and potassium dichromate (50 µg/mL) to inhibit the growth of fungi and many fast-growing Gram-negative bacteria. The agar plates were incubated at 28 °C and inspected periodically for growth for up to 8 weeks. Colonies in different morphologies were selected and re-streaked several times on TSA until colonies were visually free of contamination. The obtained pure cultures were preserved in 20% (*v*/*v*) glycerol suspensions at −80 °C.

### 2.3. Molecular Identification and Phylogenetic Analysis

Genomic DNAs were extracted from purified isolates with the Chelex-100 agent, according to Zhou et al. [48]. 16S rRNA genes were amplified with the forward primer 27f (5′-AGAGTTTGATCMTGGCTCAG-3′) and the reverse primer 1492r (5′-GGTTACCTTGTTACGACTT-3′), and the PCR reactions were performed exactly as described in our previous study [49]. The PCR products were sent to Sangon Biotech Company (Shanghai, China) for Sanger sequencing. The taxonomic affiliation of the isolates was determined by comparing their partial 16S rRNA gene sequences with those available in the NCBI GenBank (https://www.ncbi.nlm.nih.gov/genbank/ (accessed on 13 October 2021)) and EzBioCloud databases (https://www.ezbiocloud.net/identify (accessed on 15 October 2021)) [50]. Phylogenetic analysis was performed using MEGA X [51] software with the neighbor-joining algorithm based on the Kimura 2-parameter model [52]. The strength and reliability of the phylogenetic trees were evaluated by 1000 bootstrap replicates.

### 2.4. MATRIX Cultivation and Antimicrobial Activity Assay

Small-scale cultivation of the isolates was achieved in a 24-well microbioreactor cultivation system known in-lab as the MATRIX [46,47], which can provide rapid, cost-effective, and high-throughput cultivation profiling under multiple media conditions. A total of 192 isolates were selected to participate in the MATRIX cultivation. The 192 strains were selected on the basis of phylogenetic analyses of partial 16S rRNA gene sequences and a comparison of morphological characteristics. A sterile loop was used to transfer mycelia or spores from an agar plate culture to a 24-well polypropylene plate containing 2 mL of a liquid medium with a 40 mm depth and a cross section of 17 × 17 mm for each well. Four different fermentation media (FM1 to FM4, see Table S2) were used in this MATRIX cultivation system. The 24-well plates were sealed with air-permeable covers and incubated at 28 °C with shaking at 900 rpm in a gyratory shaker (Kühner AG, Basel, Switzerland) for about 7 days. After the fermentation, entire fermentation cultures were freeze-dried, and the residue in each well was extracted in situ with methanol. Briefly, the freeze-dried fermentation cultures were macerated in methanol (1 mL/well) for 30 min in an ultrasonic water bath and then centrifuged at 4500 rpm for 20 min to obtain the supernatant. The residue was re-extracted with 1 mL of methanol in the sonicating water bath, centrifuged, and the supernatant was pooled with that from the first extraction. After being extracted with methanol twice, about 1800 µL of the methanol extract from each well was obtained and transferred to 2-mL wells of a recipient plate. The methanol solvent was removed under the N_2_ stream, and the residue of each well was dissolved in 50 µL of DMSO to obtain the crude extract. All the extract plates were stored at −20 °C until assayed.

The antibacterial activities of the 768 crude extracts (192 strains × 4 media) generated by MATRIX Cultivation were evaluated using a high-throughput antibacterial assay in 96-well plates. Twelve strains of “ESKAPE” bacteria were used as indicator microorganisms: *Enterococcus faecalis* (ATCC 33186 and NO. 310682), *Staphylococcus aureus* (ATCC 29213 and ATCC 33591), *Klebsiella pneumoniae* (ATCC 10031 and ATCC 700603), *Acinetobacter baumannii* (NO. 2799 and ATCC 19606), *Pseudomonas aeruginosa* (ATCC 27853 and NO. 2774), and *Escherichia coli* (ATCC 25922 and ATCC 35218). All strains were deposited in the Institute of Medicinal Biotechnology, Chinese Academy of Medical Sciences. The test organisms were grown at 37 °C overnight in MHB (Mueller-Hinton Broth), and the growth of the cultures was quantified to an absorbance reading of 0.5 at 600 nm using a Cytation 3 microplate reader (Tecan, Sunrise, Austria) by diluting with MHB to obtain a standard inoculum equivalent to 10^5^ CFU/mL. The bacterial suspensions were then dispensed onto sterile, flat-bottomed, 96-well polystyrene microplates with a volume of 100 µL per well. Within each 96-well assay plate, four wells in the first row (Row 1) contained 100 µL of media alone, serving as a sterility check, and the rest four wells contained a standard antibiotic, levofloxacin (1 µg/mL), serving as the positive control. One microliter of each test sample (in DMSO) was added into the next ten rows (Rows 2 to 11) of wells, which contained 100 µL of bacterial suspension. The last row (Row 12) contained 100 µL bacterial culture with 1 µL DMSO serving as a negative control. Plates were then incubated at 37 °C for 24 h, and bacterial growth was measured in terms of optical density (OD) recorded at 600 nm. All experiments were replicated three times on separate plates. The inhibition rate was calculated using the formula [53]:

Inhibition % = [1 − (*t*_24_ − *t*_0_)/(*C*_24_ − *C*_0_)] × 100

where *t*_0_ is the absorbance value of a test well prior to incubation and *C*_0_ is the corresponding absorbance value of the negative control; *t*_24_ is the absorbance value of a test well after incubation at 37 °C for 24 h, and *C*_24_ is the corresponding absorbance value of the negative control well at 24 h. In this study, an inhibitory rate above 60% was defined as effective inhibition.

### 2.5. Determination of Antibacterial Mechanism

The “pDualrep2” reporter system was used to evaluate the mechanism of antimicrobial action of strain extracts with antimicrobial activity. This system is based on a hypersensitive *E. coli* stain JW5503 (Δ*tolC*) [54] transformed with the “pDualrep2” plasmid. This system allows us to sort out suppressors of protein synthesis, or SOS-response inducers. Workflow was described previously [55]. In brief, 2 µL DMSO extract of each strain was spotted on the agar plate containing a lawn of the reporter strain *E. coli* JW5503 Δ*tolC*, which was transformed by the reporter plasmid “pDualrep2”. After overnight incubation at 37 °C, the plate was scanned by the ChemiDoc Imaging System (Bio-Rad Laboratories, Hercules, CA, USA) with two channels: “Cy3-blot” (553/574 nm, green pseudocolor) for turbo red fluorescent protein (TurboRFP) fluorescence and “Cy5-blot” (588/633 nm, red pseudocolor) for Katushka2S fluorescence. Induction of expression of Katushka2S is triggered by translation inhibitors, while TurboRFP is upregulated by DNA damage-induced SOS responses. Erythromycin (Ery, 5 mg/mL, 1 µL) and Levofloxacin (Lev, 50 µg/mL, 1 µL) were used as positive controls for inhibitors of protein and DNA biosynthesis, respectively.

### 2.6. Scale-Up Cultivation and Antimicrobial Tests with Paper-Disk Diffusion Method

Strains 378 and 549 were cultivated in 500-mL Erlenmeyer flasks containing 100 mL of FM4 liquid medium at 28 °C with continuous shaking at 180 rpm for 7 days. Crude extracts were obtained after solvent extraction using ethyl acetate (EA) in an equal volume of 100 mL twice. The organic layer was condensed by rotary evaporation under reduced pressure and then dissolved in 1 mL of methanol for the following antimicrobial assay with the paper-disk diffusion method. Briefly, 50 µL of each extract was loaded on a sterile paper disk (6 mm in diameter) and dried up under ambient temperature, then the paper disk was placed on the MH (Mueller-Hinton) agar spread with the twelve “ESKAPE” strains. Positive control disks were inoculated with 10 µL of enrofloxacin (100 µg/mL), while negative control disks were inoculated with 50 µL of methanol. Antimicrobial activity was evaluated by measuring the diameter of the inhibition zone after 24 h of incubation at 37 °C.

### 2.7. UPLC/ESI-QTOF-MS/MS Analyses

EA extracts of strains were prepared in an appropriate volume of LC/MS-grade MeOH to a concentration of 1 mg/mL. UPLC-MS/MS analysis of the crude extracts was performed on an Acquity UPLC I-Class System coupled with a Xevo G2-XS QTOF Mass Spectrometer controlled by MassLynx V4.1 software (Waters, Milford, MA, USA). Samples were injected and separated on an ACQUITY UPLC BEH C_18_ column (2.1 × 100 mm, 1.7 µm, Waters, Milford, MA, USA) with an injection volume of 1 µL. A binary mobile phase system consisted of mobile phase A: 99.9% water/0.1% formic acid, and mobile phase B: 100% acetonitrile. Chromatographic separation was performed at a flow rate of 0.3 mL/min in a linear gradient of phase B from 10% to 90% for 18 min (0–18), followed by 90% B for 2 min (18–20), and back to the initial condition for 3 min. Mass spectra were acquired with an electrospray ionization (ESI) source over a mass range of *m*/*z* 100–2000 Da in the positive mode. The other parameters were set as follows: source temperature, 100 °C; desolvation temperature, 250 °C; sampling cone voltage, 40 eV; capillary voltage, 2 kV; source offset voltage, 80 eV; cone gas flow, 30 L/h; and desolvation gas flow, 600 L/h. MS/MS detection was carried out in continuum format with the mass range from 50 to 2000 Da in low collision-energy (CE) of 2 V and a high ramp CE of 40–60 V, and the scan time was 0.2 s. Solvents and non-inoculated medium extracts were also analyzed under the same settings.

### 2.8. Molecular Networking and Chemical Prediction

LC-MS/MS data were converted to mzML format and then uploaded to the Global Natural Products Social (GNPS) webserver (http://gnps.ucsd.edu (accessed on 3 November 2022)) to construct a Molecular Network (MN) [56]. Parameters were set as default except as follows: minimum cosine score, 0.6; parent mass tolerance, 0.02 Da; MS/MS fragment ion tolerance, 0.02 Da; minimum matched peaks, 4; minimum cluster size, 2. The generated networks were visualized with Cytoscape V3.9.1 [57], where nodes corresponding to peaks originating from media and solvents were deleted. The spectra in the network were searched against GNPS spectral libraries in the same manner as the input data. Molecular formula predictions were carried out with MassLynx version 4.1 for the annotation of parent ions. Predicted molecular formulae were searched against databases such as the Natural Product Atlas (NPAtlas, www.npatlas.org) [58] and StreptomeDB [59].

### 2.9. Genome Sequencing and Biosynthesis Gene Clusters (BGCs) Identification

Genomic DNAs of strains 378 and 549 were extracted following the method of Marmur [60]. The draft genomes were sequenced using the DNBSEQ platform at the Beijing Genomics Institute (Shenzhen, China). Raw reads of low quality from paired-end sequencing (those with consecutive bases covered by fewer than five reads) were discarded. The sequenced reads were assembled using SOAPdenovo version 2.04 software [61]. The whole-genome sequences of strains 378 and 549 were deposited in the NCBI GenBank under accession numbers JASKMS000000000 and JASKMT000000000, respectively, and were annotated by the NCBI prokaryotic genome annotation pipeline (PGAP) [62]. AntiSMASH (version 7.0) was used to predict the biosynthetic gene clusters for the production of secondary metabolites [63].

## 3. Results

### 3.1. Taxonomy and Biodiversity of Cultivable Actinobacteria

**Figure 2:**
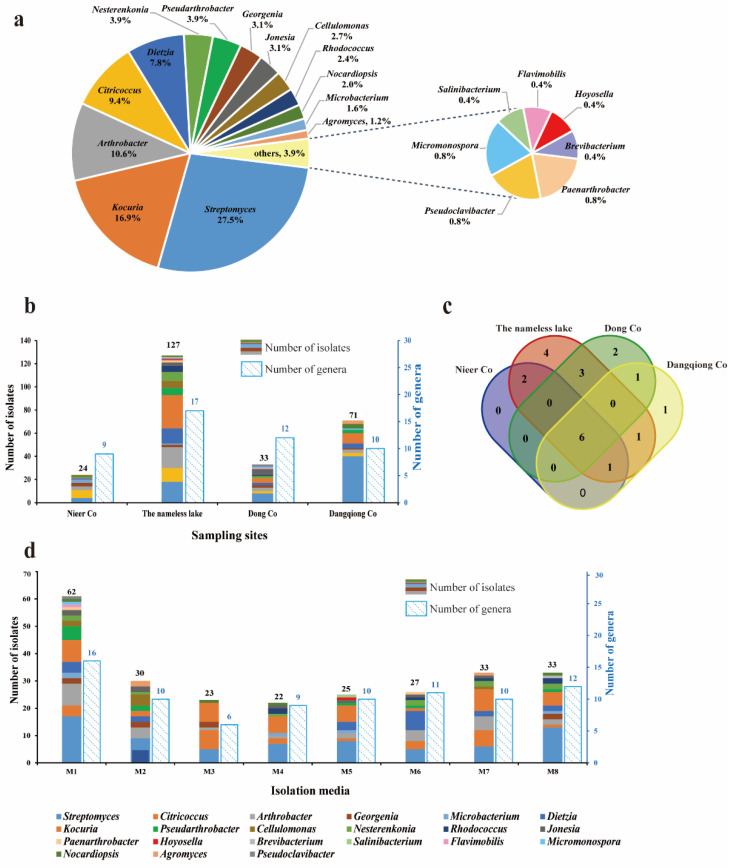
Diversity of cultivable actinobacteria recovered from the sediment samples of four saline lakes. (**a**) A pie chart showing the percentage of recovered isolates affiliated with 21 different genera. (**b**) Numbers of actinobacteria in different genera isolated from four saline lakes. (**c**) Venn diagram showing the number of genera common and unique distributed in the four saline lakes. (**d**) Numbers of actinobacteria in different genera isolated from eight culture media (M1–M8). The compositions of the eight culture media (M1–M8) are shown in Supplementary Table S2.

A total of 255 actinobacterial strains were isolated from the sediment samples of four saline lakes. These isolates were phylogenetically affiliated to 21 genera, 12 families, and 7 orders based on the 16S rRNA gene sequence analysis (Table S3). Among the 255 isolates, genera *Streptomyces* (70 isolates) and *Kocuria* (43 isolates) were numerically dominant, followed by *Arthrobacter* (27), *Citricoccus* (24), *Dietzia* (20), *Pseudarthrobacter* (10), *Nesterenkonia* (10), *Georgenia* (8), *Jonesia* (8), *Cellulomonas* (7), *Rhodococcus* (6), *Nocardiopsis* (5), *Microbacterium* (4), *Agromyces* (3), *Paenarthrobacter* (2), *Pseudoclavibacter* (2), and *Micromonospora* (2), as well as an additional four genera that were represented by only one isolate (Figure 2a). The similarity index of the 16S rRNA gene sequence was obtained from BLAST analysis on the EzBioCloud server [50]. Among all the isolates, 34 strains displayed 100% similarity with the published type strains in the phylum *Actinomycetota* [64], 181 strains exhibited 99–100% similarity, and 39 strains exhibited 98.65–99% similarity. Only one strain, LD-9, isolated from the Dong Co sediment, exhibited the highest 16S rRNA gene sequence similarity of 98.4% towards the type strain of *Pseudoclavibacter triregionum* [65], indicating that LD-9 could represent a novel taxon within the family *Microbacteriaceae*. A phylogenetic tree based on the analysis of the 16S rRNA gene sequence of 37 type species of *Microbacteriaceae* (Figure S1) showed that strain LD-9 grouped with the members of the genus *Pseudoclavibacter* and fell into a coherent subclade with *Pseudoclavibacter triregionum*, indicating that strain LD-9 should represent a novel species of the genus *Pseudoclavibacter*. Further polyphasic studies will be performed to determine the taxonomic position of strain LD-9.

Cultivable Actinobacteria diversity retrieved from the four saline lakes is illustrated in Figure 2b. The sample from the nameless saline lake yielded the most notable richness and recoverability of actinomycetes, with 127 strains distributed in 17 genera, while the sample from Nieer Co. gave the lowest, with 24 strains distributed in 9 genera. A Venn diagram illustrated the number of genera common and unique distributed in the four saline lake sediments (Figure 2c). Six genera, including *Streptomyces*, *Kocuria*, *Arthrobacter*, *Citricoccus*, *Dietzia*, and *Georgenia*, were recovered from all sampling locations; Genera *Cellulomonas* and *Microbacterium* were present in both Nieer Co. and the nameless saline lake; Genus *Micromonospora* were present in both Dong Co. and Dangqiong Co. Sediment samples from different saline lakes also had exclusive genera. Four unique genera were only retrieved from the nameless saline lake, corresponding to *Hoyosella*, *Brevibacterium*, *Paenarthrobacter*, and *Salinibacterium*; Genera *Flavimobilis* and *Pseudoclavibacter* were only harvested from Dong Co; Genus *Agromyces* was only harvested from Dangqiong Co. Furthermore, the respective genus abundances were revealed to be considerably different among sampling sites. *Streptomyces* represented the major genus in both Dangqiong Co. and Dong Co. with 56.3% and 24.2%, respectively. *Kocuria* was the most abundant genus (22.8%) in the nameless salty lake, while *Citricoccus* was the most abundant genus (29.1%) in the Nieer Co.

To improve the diversity of actinobacteria and mine more rare species, eight isolation media with different carbon and nitrogen sources, salinity levels, and pH values were applied (Table S2). Our results revealed that medium composition had a noticeable impact on the recovery of isolates (Figure 2d). The modified PYG medium (M1, pH 8.0) provided the highest recoverability and diversity, yielding 62 isolates from 16 different genera, while the modified R2A medium (M3, pH 8.0) exhibited the lowest diversity, with only 23 strains from 6 genera. Genera *Streptomyces*, *Kocuria*, and *Arthrobacter* were recovered from all eight media, whereas *Flavimobilis* was uniquely present in the modified PYG medium (M1, pH 8.0), and *Hoyosella* and *Salinibacterium* were isolated only from the alkaline modified PYG medium (M5, pH 10.0). To isolate halotolerant bacteria from saline lakes, three concentrations of NaCl (0%, 3%, and 5%, *w*/*v*) were supplemented into the isolation media. We obtained a total of 144 strains from 20 different genera in media without NaCl (M1, M2, M5, and M6), 56 strains from 12 genera in media with 3% NaCl (M3 and M7), and 55 strains from 15 genera in media with 5% NaCl (M4 and M8). Genera such as *Streptomyces*, *Nocardiopsis*, *Nesterenkonia*, and *Kocuria*, which are known to contain high numbers of halotolerant or halophilic actinobacteria, were mostly found in media with 5% NaCl.

### 3.2. MATRIX Cultivation and Antibacterial Activity

**Figure 3:**
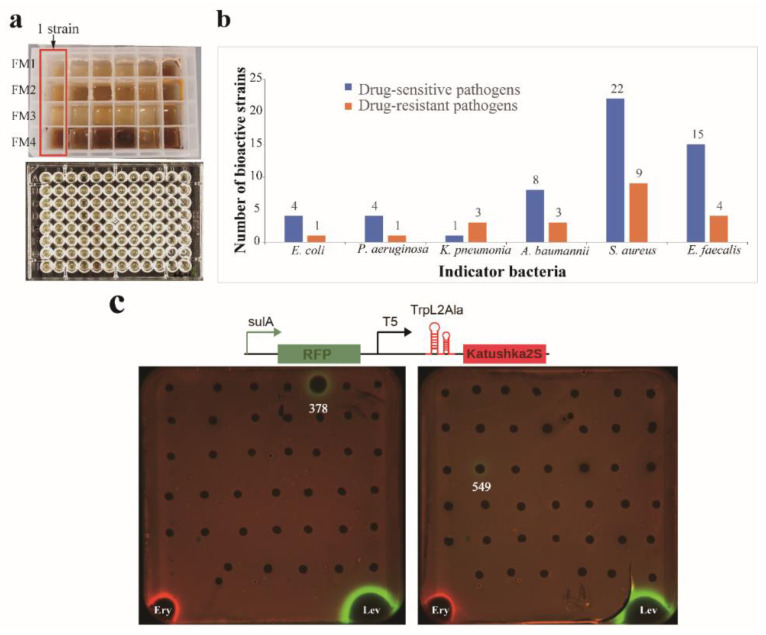
Assays of antibacterial activity and mechanism of action. (**a**) Multiple actinobacteria were cultivated in a miniaturized 24-well microbioreactor system (MATRIX cultivation), and the antibacterial activity was evaluated by using a turbidometric screening assay in 96-well plates. (**b**) Antibacterial profile of the tested actinobacteria against twelve “ESKAPE” pathogens. (**c**) The dual-fluorescent reporter system “pDualrep2” is sensitive to protein synthesis inhibitors or SOS-response inducers. Translation inhibitors trigger the induction of Katushka2S (red pseudocolor) expression and TurboRFP (green pseudocolor) expression on DNA damage-caused SOS responses. Ery, Erythromycin; Lev, levofloxacin.

The OSMAC (One Strain Many Compounds) approach is a simple and effective technique to enhance the chemical diversity of microbial metabolites. Traditional methods of microbial biodiscovery involve small-scale liquid cultivations in shake flasks (10–100 mL), which can be cumbersome, time-consuming, and costly when dealing with large numbers of microbial isolates [66]. In this study, we utilized a miniaturized 24-well microbioreactor system called “MATRIX cultivation” [46,47] to facilitate OSMAC fermentation (Figure 3a). This allowed us to cultivate 192 isolates in arrays of 4 different liquid media compositions, yielding 768 crude extracts through in situ solvent extraction on individual MATRIX culture wells. The antimicrobial activity of these extracts was then evaluated by using a turbidometric screening assay monitored in OD_600_ in 96-well plates, with twelve “ESKAPE” strains used as indicator pathogens. An inhibitory rate above 60% was considered positive for antimicrobial activity. The results showed that out of the 192 tested strains, extracts from 46 strains exhibited antagonistic activity against at least one of the tested pathogens (Table S4). These bioactive strains were affiliated with 13 genera, including *Streptomyces* (14 strains), *Arthrobacter* (8), *Kocuria* (7), *Nesterenkonia* (4), *Dietzia* (3), *Pseudarthrobacter* (2), *Nocardiopsis* (2), *Agromyces* (2), *Georgenia* (1), *Cellulomonas* (1), *Jonesia* (1), *Micromonospora* (1), and *Hoyosella* (1). The percentage of bioactive strains against different test pathogens is given in Figure 3b. Inhibitory activity was most frequently observed against the drug-sensitive *Staphylococcus aureus* (22 isolates), while bioactive strains against the drug-resistant bacteria *Escherichia coli* and *Pseudomonas aeruginosa* were the least frequent (1 isolate). Among the four media (FM1 to FM4, see Table S2) employed in MATRIX cultivation, 23 isolates exhibited antibacterial activity in media FM4, 17 isolates in FM1, 7 isolates in FM2, and 7 isolates in FM3. These results indicated that the FM4 medium was the optimum medium for producing bioactive components.

### 3.3. Assay of Antibacterial Mechanism Action

To distinguish bioactive strains with varying antibacterial mechanisms, MATRIX extracts of the 46 bioactive strains (Table S4) were assayed using a double fluorescent protein reporter (pDualrep2) system. This system has already succeeded in the early identification of antibacterial inhibitors that may block bacterial translation or disrupt DNA replication [67,68,69]. The findings, as shown in Figure 3c, indicate that *Streptomyces* strains 378 and 549 triggered an SOS response and induced the expression of the TurboRFP. We suggest that these strains can produce compounds that cause DNA damage, similar to levofloxacin. None of the strain extracts could induce the expression of Katushka2S, implying that no extract of the strains could trigger translation stalling on the ribosome and inhibit protein biosynthesis, which is similar to erythromycin. Based on the displayed activity, *Streptomyces* 378 and 549 have garnered particular interest for scaling up and conducting chemical studies on their bioactive components.

### 3.4. Scale-up Cultivation and Antimicrobial Activity Revaluation of Strains 378 and 549

BLAST analysis of the 16S rRNA gene sequences from strains 378 and 549 on the EzBioCloud server revealed that they belonged to the *Streptomyces* genus. The nearly full-length 16S rRNA gene sequence of strain 378 (GenBank NO. OQ509818, 1397 bp) shared the highest sequence similarity (99.4%) with *Streptomyces marokkonensis* Ap1^T^; strain 549 (GenBank NO. OQ509816, 1425 bp) showed the highest identity (99.9%) to *Streptomyces sparsus* YIM 90018^T^. In contrast to MATRIX-cultivation, the fermentation broth of the two strains was separately prepared in a 500-mL Erlenmeyer flask with 100 mL of liquid medium. The antimicrobial activity of the EA extracts was evaluated through the paper-disk diffusion method and presented as inhibition zone diameters against the ESKAPE strains. The antimicrobial activities of the two strains cultivated in 100 mL of liquid medium are presented in Table S5. Strains 378 and 549 exhibited potent antimicrobial activities against all target gram-positive bacteria, and 549 displayed some activity against the gram-negative bacteria *Escherichia coli* and *Acinetobacter baumannii*. These results are consistent with the activities previously observed in the MATRIX-cultivation assay. Some differences observed may be due to the different solvents used for extraction and variations in cultural conditions, such as rotation speed and oxygen quantity.

### 3.5. Chemical Analysis of Crude Extracts from Strains 378 and 549

Modern mass spectrometry methods allow not only the general analysis of secondary metabolites but also the possibility for the identification of individual compounds using information from pre-existing databases [70]. Secondary metabolites present in the crude extracts of strains 378 and 549 were subjected to UPLC-QTOF MS/MS analysis. The acquired UV spectra, molecular formulae, and accurate masses were searched against multiple databases. The identified compounds produced by the two strains are summarized in Table S6.

**Figure 4:**
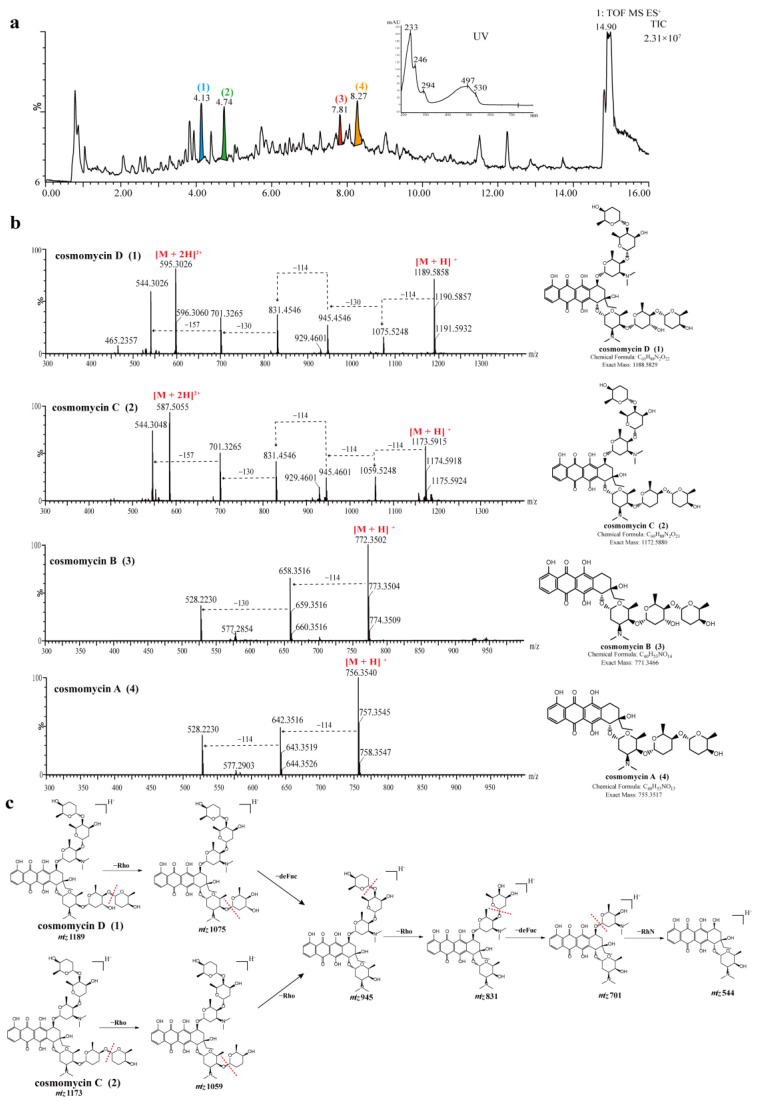
UPLC-HR-MS/MS spectra and identification of bioactive compounds from the EA extract of strain 378. (**a**) Total ion chromatogram (TIC) of extracts from strain 378, and four major compounds (peak 1–4) were labeled in the TIC. (**b**) MS/MS spectra of four identified compounds (**1**–**4**). (**c**) The annotated fragmentation patterns of peak 1 (cosmomycin D) and 2 (cosmomycin C).

The UPLC-UV-MS/MS chromatogram of strain 378 showed that there were a series of spectrally similar compounds with a characteristic maximum UV absorption at about 497 nm (Figure 4a). These compounds were tentatively identified as anthracycline-type compounds by searching databases and comparing the UV spectral data with the literature references. Out of these compounds, four prominent peaks (**1**–**4**) were noted with chromatographic retention times (*t*_R_) of 4.13 min, 4.74 min, 7.81 min, and 8.27 min, which presented mass spectra with [M + H]^+^ ions at mass-to-charge ratios (*m*/*z*) of 1189.5878, 1173.5925, 772.3516, and 756.3598, respectively. Based on their spectral properties and comparing them to literature data, they were putatively identified as cosmomycin D (**1**), cosmomycin C (**2**), cosmomycin B (**3**), and cosmomycin A (**4**), respectively. Moreover, the characteristic UV/Vis absorption bands at about 233, 246, 294, 497, and 530 nm for compounds **1**–**4** were consistent with those of previously reported cosmomycins [71,72]. Their structures were also unambiguously confirmed by comparison of the precursor ion *m*/*z* value and fragmentation pattern with reference data [73,74]. For example, the MS2 spectrum of cosmomycin D displayed a positive ion in high abundance at *m*/*z* 595.3 [M + 2H]^2+^ and another in lower abundance at *m*/*z* 1189.6, corresponding to the molecular ion [M + H]^+^. The dissociation products from cosmomycin D were: 1075.5 [–114, corresponding to the loss of the outermost sugar, rhodinose (rho) in one trisaccharide chain]; 945.3 [–130, corresponding to the loss of a middle sugar, 2-deoxy-L-fucose (defuc)]; 831.3 [–114, corresponding to the loss of the outermost sugar (rho) in the second trisaccharide chain]; 701.3 [–130, corresponding to the loss of the middle sugar (defuc) from the second chain]; and 544.3 [–157, corresponding to the loss of the innermost amino sugar, rhodosamine (RhN)]. The dissociation products from 1173.3 for cosmomycin C were 1059.1 (–114) → 945.1 (–114) → 831.2 (–114) → 701.2 (–130) → 544.3 (–157). Cosmomycin C revealed a similar fragmentation pathway with cosmomycin D, except for the first product, 1059.1 (–114), which was in correspondence with the middle sugar in one trisaccharide chain lacking a hydroxyl group. It is worth noting that cosmomycins have been previously reported to possess antibacterial activities against gram-positive bacteria, show significant antitumor activity against human tumors, and can induce the differentiation of Friend leukemia cells [75,76]. A recent study has also suggested that cosmomycin A inhibits the growth of *Plasmodium falciparum* 3D7 cells with an IC_50_ value of 2.8 nM [77].

**Figure 5:**
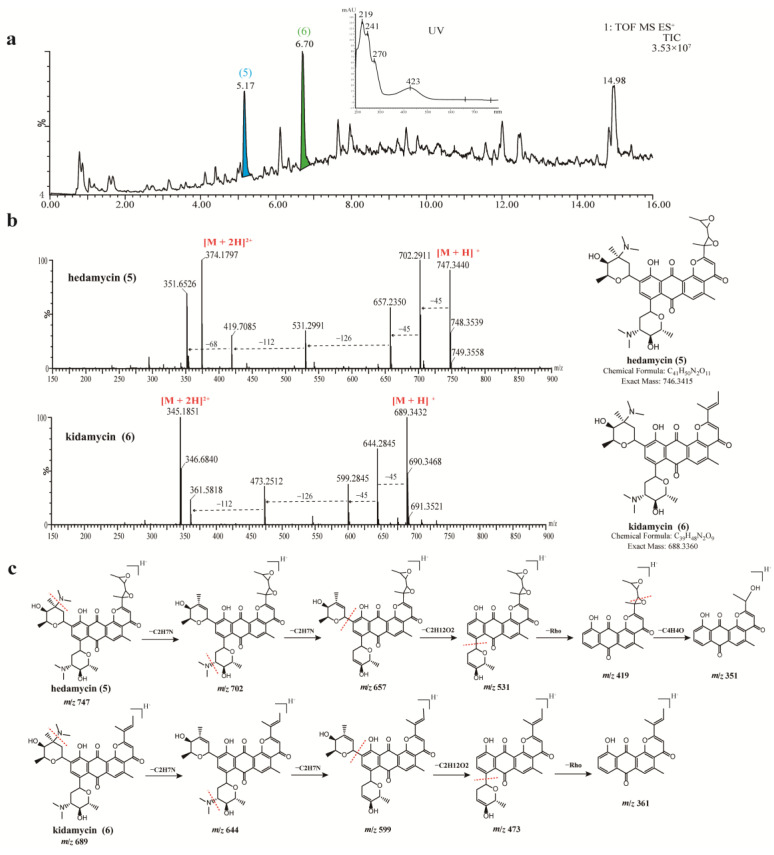
UPLC-HR-MS/MS spectra and identification of bioactive compounds from the EA extract of strain 549. (**a**) Total ion chromatogram (TIC) of extracts from strain 549, and two major compounds (peak 5–6) were labeled in the TIC. (**b**) MS/MS spectra of two identified compounds (**5**–**6**). (**c**) The annotated fragmentation patterns of peaks 5 (hedamycin) and 6 (kidamycin C).

Antibacterial components present in the crude extract of strain 549 were analyzed in the same manner. A series of compounds with similar spectra and molecular weights ranging from 660 to 750 were detected, all exhibiting a distinct UV absorption peak at around 423 nm (Figure 5a). Upon searching in the database, two antibiotics belonging to the angucycline family were annotated: hedamycin at *t*_R_ 5.17 min (Peak **5**, *m*/*z* 747.3470 [M + H]^+^) and kidamycin at t*_R_* 6.70 min (Peak **6**, *m*/*z* 689.3422 [M + H]^+^). The structures of compounds **5** and **6** were unambiguously verified by comparisons of the specific MS/MS fragmentation pattern (Figure 5c). Hedamycin and kidamycin share similar structures, each containing two amino deoxyhexoses: an anglosamine sugar attached to the C8 and a N,N-dimethylvancosamine attached to the C10 via two unusual C-glycoside bonds. The difference between them lies in the substituent at C2: kidamycin features an olefinic residue, while in hedamycin, the subgroup is substituted with a bisepoxide [78,79]. Hedamycin and kidamycin are known for their broad-spectrum antibacterial activity as well as their insecticidal, anthelmintic, larvicidal, and coccidiostatic properties [80,81,82,83].

### 3.6. Molecular Networking Analysis of Streptomyces 378 and 549

**Figure 6:**
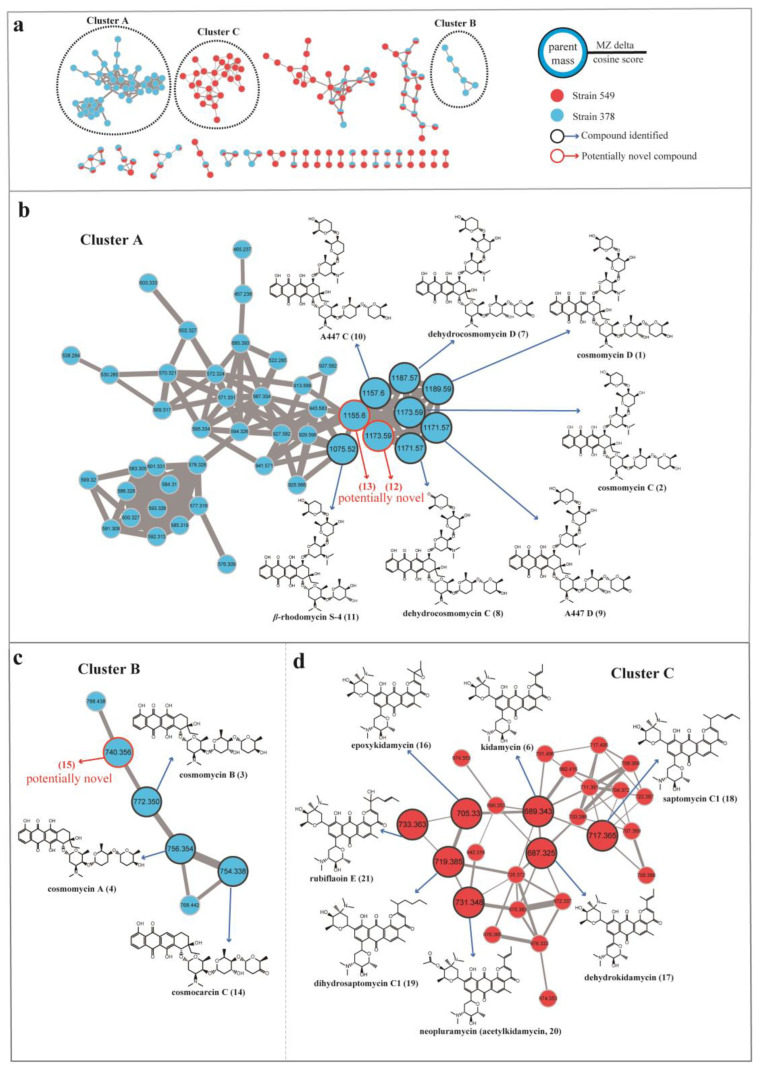
Molecular network for annotation of secondary metabolites from EA extracts from strains 378 and 549. The molecular network generated by LC-MS/MS data were analyzed via the GNPS platform. (**a**) Three annotated anthracycline clusters (A, B, and C) in the molecular network of strains 378 and 549 Each node represents the *m*/*z* value of the parent ion, and the edge thickness signifies cosine score similarity. (**b**) Observation of molecular cluster A allows highlighting annotation of derivatives of cosmomycin C and D in strain 378. (**c**) Observation of molecular cluster B allows highlighting annotation of derivatives of cosmomycin A and B in strain 378. (**d**) Observation of molecular cluster C allows highlighting annotation of derivatives of kidamycin in strain 549.

To facilitate the dereplication of known compounds and further explore novel aromatic polyketide antibiotics, we employed a molecular networking approach based on MS/MS data acquired from *Streptomyces* 378 and 549 by using a GNPS-based computational workflow. This approach enabled us to construct a molecular network consisting of 173 nodes that covered a broad range of molecules from *m*/*z* 300 to 1200 (Figure 6). Each node within this network represented a molecule, displaying the molecular mass of the parent ion, fragment ion, or different adduct masses. Hence, it was possible to find several nodes corresponding to the same compound. Through elaborate inspection of the resulting molecular network, we were particularly intrigued by three clusters (Clusters A, B, and C) containing precursor masses matching the identified aromatic polyketides. Molecules present in Clusters A and B corresponded to the cosmomycin analogues in strain 378, and molecules present in Cluster C corresponded to the kidamycin analogues in strain 549.

In cluster A, a large number of nodes clustered with the previously described molecules cosmomycin C (*m*/*z* 1173.5925, [M + H]^+^) and cosmomycin D (*m*/*z* 1189.5878, [M + H]^+^), indicating the presence of several additional derivatives in strain 378. Analysis of the differences in atomic mass units (amus) relative to the two annotated nods led to the putative identification of five analogs of cosmomycin C or D, including dehydrocosmomycin D (*m*/*z* [M + H]^+^ 1187.5710, C_60_H_87_N_2_O_22_, compound **7**) [84], dehydrocosmomycin C (*m*/*z* [M + H]^+^ 1171.5754, C_60_H_87_N_2_O_21_, **8**) [85], A447 D (*m*/*z* [M + H]^+^ 1171.5754, C_60_H_87_N_2_O_21_, **9**) [86], A447 C (*m*/*z* [M + H]^+^ 1157.5942, C_60_H_89_N_2_O_20_, **10**) [86], and *β*-rhodomycin S-4 (*m*/*z* [M + H]^+^ 1075.5168, C_54_H_79_N_2_O_20_, **11**) [87]. The annotation of these five molecules was supported by a comparison of mass fragmentation patterns and isotope distributions with those previously reported (Figure S2). Compound **12** (*m*/*z* [M + H]^+^ 1173.5925, C_60_H_89_N_2_O_21_**)** possessed an identical molecular mass with cosmomycin C but gave a different fragmentation pattern and retention time in HPLC, indicating that it should be a structural isomer of cosmomycin C. Collisionally-activated dissociation of the precursor ion (1173.6) of compound **12** released fragments at 1059.1(–114) → 929.3(–130) → 815.4(–114) → 685.3(–130) → 528.3(–157). This dissociation pattern is consistent with compound **12** bearing the same sugar residues as cosmomycin D but containing one fewer hydroxyl group in the aglycone. Compound **13** (*m*/*z* [M + H]^+^ 1155.5762, C_60_H_87_N_2_O_20_) was postulated to contain a double bond in one outermost sugar (rho), as compared to A447 C (**10**), owing to the disparity in molecular formula (2 Da) and comparable fragmentation pattern. No matches were retrieved from the database for compounds **12** and **13**, thereby implying they may represent novel derivatives of cosmomycins.

Cluster B, consisting of 6 nodes with precursor masses ranging from *m*/*z* 740 to 788 Da, was identified as the family of cosmomycin A (**4**, *m*/*z* 756.3598 [M + H]^+^) and cosmomycin B (**3**, *m*/*z* 772.3516 [M + H]^+^). Through propagated annotation of the connected nodes, two additional derivatives were discovered. Compound **14** (*m*/*z* [M + H]^+^ 754.3417, C_40_H_52_NO_13_) with a direct linkage to the node of cosmomycin A was putatively identified as cosmocarcin C [88]. Compound **15** was inferred to be dehydroxylated cosmomycin A with a [M + H]^+^ ion at *m*/*z* 740.3627 that displayed a neutral loss of oxygen (16 Da). Despite a thorough search within the database, no match was found for compound **15**, indicating it is possibly a novel compound. However, these potential novel compounds of the cosmomycin family were only detected in trace amounts during the examination of the original chromatogram data of strain 378.

In the molecular network of the kidamycin family (Cluster C, depicted in Figure 6d), the protonated molecular ion of kidamycin (**6**) was found to be clustered with 25 nodes with precursor masses ranging from *m*/*z* 642 to 733 Da. Starting with the kidamycin, six additional derivatives of kidamycin were identified, including the 14,16-epoxy analogue of kidamycin (epoxykidamycin, *m*/*z* [M + H]^+^ 705.3298, C_39_H_49_N_2_O_10_, compound **16**) [89], dehydrokidamycin (*m*/*z* [M + H]^+^ 687.3245, C_39_H_47_N_2_O_9_, **17**), saptomycin C1 (*m*/*z* [M + H]^+^ 717.3750, C_41_H_53_N_2_O_9_, **18**) [90], dihydrosaptomycin C1 (*m*/*z* [M + H]^+^ 719.3541, C_41_H_55_N_2_O_9_, **19**) [91], 3”-O-acetylkidamycin (neopluramycin, *m*/*z* [M + H]^+^ 731.3520, C_41_H_51_N_2_O_10_, **20**) [92] and rubiflaoin E (*m*/*z* [M + H]^+^ 733.3668, C_41_H_53_N_2_O_10_, **21**) [93]. UPLC-MS/MS spectra of these putative kidamycin derivatives (**16**–**21**) are illustrated in Figure S3.

### 3.7. Identification of Biosynthetic Gene Clusters (BGCs) for Strains 378 and 549

The draft genome sequences of strains 378 and 549 were analyzed via the bioinformatic tool antiSMASH7.0 to determine their putative biosynthetic capabilities. For stain 378, a total of 30 putative BGCs were detected, including three types of polyketide synthases (T1PKS, T2PKS, and T3PKS), non-ribosomal peptide synthase (NRPS), class i-lanthipeptides, butyrolactone, etc. (Table S7). Among the 30 identified BGCs, six clusters were annotated as orphan BGCs for which no homologous gene clusters could be matched, suggesting that they possibly biosynthesize novel natural products or compounds with no characterized BGCs. As for strain 549, there were 20 BGCs predicted, and they were involved in T1PKS, T2PKS, NRPS, class i, iii-lanthipeptides, etc. (Table S8). Five out of 20 identified BGCs belonged to the known clusters encoding for ectoine, AmfS, geosmin, desferrioxamine, and lagmycin with 100% similarity, and two clusters were annotated as orphan BGCs. Notably, both strains 378 and 549 were predicted to harbor a putative T2PKS BGC, which showed high similarity to several known anthracycline-type and angucycline-type BGCs, respectively, consistent with the metabolomic analysis.

**Figure 7:**
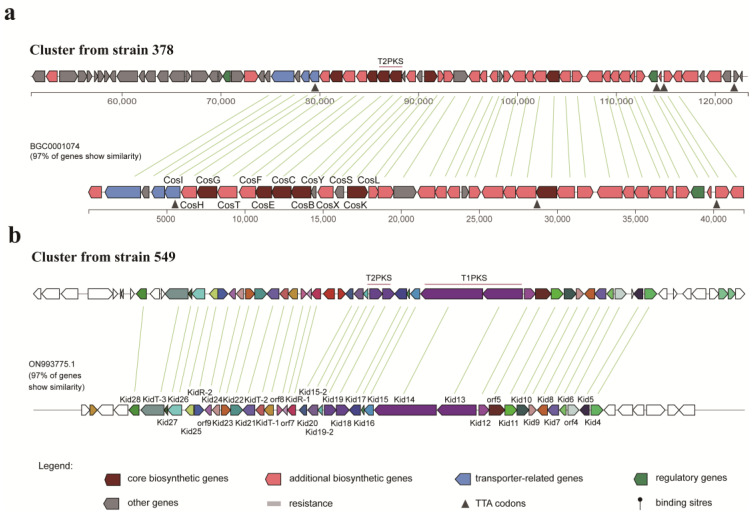
Comparison of the putative biosynthetic gene clusters from strains 378 (**a**) and 549 (**b**) with the cosmomycin D cluster (BGC0001074) and the kidamycin cluster (ON993775.1), respectively.

As outlined above, strain 378 was found to produce cosmomycins that belong to the anthracycline group. AntiSMASH analysis revealed that strain 378 harbors a predicted T2PKS BGC, which exhibits 84% similarity with the cytorhodin BGC from *Streptomyces* sp. SCSIO 1666, 97% with cosmomycin D BGC and 92% with cosmomycin B BGC from *Streptomyces olindensis*, 76% with cosmomycin C BGC from *Streptomyces* sp. CNT302, 85% with cinerubin B BGC from *Streptomyces* sp. SPB074, and 91% with rhodomycin BGC from *Streptomyces purpurascens* (Figure S4). All these compounds share a common tetracyclic benz[*b*]anthraquinone aglycone belonging to the anthracycline group. The putative T2PKS BGC from strain 378 spans about 72 Kb of contiguous genomic DNA and contains 68 genes. Three genes from locus tag ctg1_80 to ctg1_82 encode the typical minimal PKS composed of ketoacyl synthase (KS), a chain length factor (CLF), and an acyl carrier protein (ACP), which starts with a propionyl-CoA, as well as a gene at ctg1_79 encodes the putative propionyl-CoA: ACP acyltransferase, all of which is responsible for the C-21 backbone formation. In addition, there are three genes at ctg1_92, ctg1_95, and ctg1_105 encoding cyclases, three genes at ctg1_94, ctg1_96, and ctg1_110 encoding ketoreductase, three genes at ctg1_102, ctg1_104, and ctg1_106 encoding methyltransferse, one gene at ctg1_101 encoding monooxygenase, three genes at ctg1_77, ctg1_86, and ctg1_98 encoding glycosyltransferase genes, and a series of genes responsible for the generation of sugar units, all of which are involved in the tailoring process of bioactive metabolites from strain 378. As shown in Figure 7a, 97% of genes from the cosmomycin D BGC show similarity with the genes of the cluster from strain 378 and are in a similar organization, hinting that a great potential exists in the cluster from strain 378 to produce cosmomycin analogues.

Strain 549 was found to be capable of producing hedamycin and kidamycin, both of which belong to the angucycline group. AntiSMASH analysis revealed the presence of a T2PKS BGC in strain 549, which exhibited 84% similarity with the hedamycin BGC from *Streptomyces griseoruber* (Figure S5). However, several divergences in synteny were observed, prompting a further BLAST search in GenBank to identify a more closely related BGC. Fortunately, a kidamycin BGC from *Streptomyces* sp. strain W2061 was discovered, displaying 97% similarity with the putative BGC from strain 549 and sharing a similar synteny (Figure 7b). This finding suggests that the T2PKS BGC in strain 549 has a high potential for producing kidamycin analogues. Different from the cosmomycin cluster in strain 378, the putative T2PKS BGC in strain 549 is a hybrid of T2PKS and T1PKS and contains 58 genes distributed in about 71 Kb of contiguous genomic DNA. According to the prediction generated by antiSMASH, the T1PKS modules located at the locus tags ctg1_37 and ctg1_38 are in charge of the generation of the starter unit in the T2PKS processing. According to the detailed gene organization, 2-methyl-2-butyryl CoA is deduced to be the most likely starter unit, while hexadienyl CoA is also a possible one, both of which are the starter units of kidamycins and hedamycins. For the T2PKS, the minimal PKS of KS and CLF at ctg1_32 and ctg1_33, respectively, is in charge of the C-23/C-24 backbone formation after 9 rounds of chain extension. In addition, there are two putative genes at ctg1_30 and ctg1_36 encoding cyclases, one gene at ctg1_29 encoding ketoreductase, three genes at ctg1_18, ctg1_43, and ctg1_50 encoding methyltransferse, one gene at ctg1_41 encoding monooxygenase, two genes at ctg1_21 and ctg1_45 encoding glycosyltransferase genes, and a series of genes responsible for the generation of sugar units, all of which are involved in the tailoring process of bioactive metabolites from strain 549.

Regarding the other biosynthetic gene clusters that were identified through antiSMASH, we were unable to designate any other secondary metabolites to the anticipated clusters. This could be attributable to most of the BGCs present in strains 378 and 549 being silent, which means the corresponding gene clusters may not have been expressed under the tested conditions. Comprehensive optimization of the culture conditions for these strains can be conducted based on this research in order to delve deeper into their metabolic synthesis potential and discover more types of active natural products.

## 4. Discussion

Over the past two decades, investigations on the community structures of archaea and bacteria within saline lakes on the Northern Tibetan Plateau have primarily relied on culture-independent molecular methodologies [94,95,96]. Only three pieces of literature have reported the recovery of cultivable actinobacteria from saline lakes in this region [97,98,99]. Furthermore, earlier studies have highlighted notable differences in bacterial community composition across the Tibetan Plateau at different zones [100,101]. Local geochemical features such as salinity (including salinity-related major ions), pH, and total nitrogen (TN), as well as the geographical distance, were primary factors influencing the variation of bacterial community structures [95,100,101]. In the present study, we collected samples from four distinct saline lakes on the Northern Tibetan Plateau, each separated by considerable distances ranging from 40 to 684 km and situated far away from previously reported saline lakes. The observed heterogeneous biodiversity could be attributed to variations in salinity and pH levels (as shown in Table S1) among different locations. However, the precise mechanisms by which these environmental factors influence the species composition of actinobacteria in these lakes remain unclear. This intriguing subject merits further investigation to deepen our understanding of the intricate relationships between environmental conditions and actinobacterial communities within these unique saline lake ecosystems.

It is quite essential to utilize various media for bioprospecting the untapped actinobacterial resources. In the isolation process, eight different culture media were applied, and the modified PYG agar medium (M1) showed the highest recoverability and genera diversity. This finding suggests that M1 is suitable for isolating actinobacteria from saline lake sediments. Supplementing betaine and glycerol in M1 may enhance the microbial antioxidative defense system against salinity stress and contribute to better isolation of rare actinomycetes, as demonstrated in other studies [102,103]. To recover more halotolerant strains, three concentrations of NaCl (0%, 3%, and 5%, *w*/*v*) were added to the isolation media. Interestingly, a large number of actinobacteria were isolated using media without NaCl. Possible reasons for this inconsistency could be due to the fact that many halotolerant actinobacteria can tolerate a wide range of salinities and grow well in the absence of NaCl, or that the surface layer of sediment samples has a higher salinity than other parts of the sample. In the MATRIX cultivation step, inhibition activities were observed more frequently in the medium FM4. Medium FM4 contained versatile nitrogen sources such as peptone, yeast extract, and soybean meal, with soybean meal as a slowly-utilizing nitrogen source contributing to prolonged secretion periods and increased yield of antibiotic products [104]. Additionally, the addition of 4% NaCl (*w*/*v*) to the FM4 medium may stimulate the growth and metabolism of actinobacteria in the saline environment.

Multi-omics approaches have recently made big strides toward the effective exploration of microorganisms, accelerating the discovery of new bioactive compounds. In this research, bioactive components from two *Streptomyces* strains were comprehensively analyzed with multi-omics strategies. Two types of metabolites belonging to the aromatic polyketide family were unambiguously identified, including anthracyclines (cosmomycin) and angucyclines (kidamycin and hedamycin). The crude fractions extracted from both strains 378 and 549 exhibited an orange-red/red-purple color, displaying absorption within the visible light range of 400–500 nm. The chromophore responsible for this distinctive coloration originates from the tetracyclic anthracycline aglycone structure. This unique visible absorption spectrum can serve as a distinguishing marker or probe for the screening of anthracycline and angucycline compounds. Notably, the bacteriostatic effects of cosmomycin, kidamycin, and hedamycin have been reported to arise from their ability to intercalate with DNA in major and minor grooves, inducing DNA breaks via topoisomerase II poisoning [105,106,107,108]. This finding is in accordance with the fluorescence results observed in strains 378 and 549 in the dual-reporter pDualrep2 assay.

Interestingly, these aromatic polyketide antibiotics may have a potential biological function in the life cycle of their producing strains inhabiting adverse environments, such as hypersaline environments. Under poor nutritional conditions, the vegetative or substrate mycelia of *Streptomyces* are inclined to be dismantled by programmed cell death (PCD)-like mechanisms, thereby releasing nutrients to accelerate the buildup of the aerial hyphae [109]. This phenomenon coincides with the accumulation of the anthracyclines that target DNA and protect the nutrient pool from being accessed by other motile competitors [110,111]. On the other hand, cosmomycin, hedamycin, and kidamycin are all optically active dyes with an orange-red/red-purple color. Studies have demonstrated that pigments such as carotenoids, phenazines, quinones, indigoidines, and melanins generally possess antioxidative and ultraviolet-resistant properties that protect bacteria from the detrimental effects of reactive oxygen species and ultraviolet radiation [112,113]. Therefore, it is estimated that these anthracycline and angucycline compounds can offer the producing strains enhanced resistance to oxidative stress and UV radiation on the high-altitude Qiangtang Plateau.

## 5. Conclusions

The current investigation has demonstrated that the saline lakes located on the Northern Tibetan Plateau represent a promising source of diverse actinobacterial strains for the production of bioactive metabolites. By implementing combinational strategies such as MATRIX cultivation, dual-fluorescent reporter assays, UPLC-MS/MS-based metabolomic analysis, molecular networking, and genome mining, we have successfully identified the aromatic polyketide antibiotics accountable for the antibacterial activities exhibited by two *Streptomyces* strains. Furthermore, an in-depth analysis of the metabolomic data leads to the detection of some potentially novel anthracycline compounds, which highlights the effectiveness of metabolomics profiling and molecular networking in guiding the discovery of novel bioactive candidates. This study not only provides pioneering insights into the pharmaceutical potential of actinobacteria associated with saline lakes on the Northern Tibetan Plateau but also presents a practical approach for the efficient chemical dereplication of the complicated secondary metabolites produced by microorganisms.

It is also important to acknowledge that this study has several limitations that warrant consideration. Firstly, the findings of this study are based on a limited number of sediment samples collected from four specific saline lakes located on the Northern Tibetan Plateau. Fluctuations in bacterial communities across different sampling locations and seasons are not investigated in this study. The generalizability of these findings to other geographical regions or different types of saline lakes on the Northern Tibetan Plateau remains uncertain. Secondly, although the OSMAC strategy is employed in this study, the selected culture conditions are far from exhaustive. To comprehensively explore the synthetic metabolic potential of the isolated strains, alternative strategies can be pursued. These may include the adjustment of salt ion composition and concentration in the culture medium, as well as the incorporation of various small-molecule compatible solutes, enzyme inhibitors, or growth factors. Thirdly, while this study detects potentially novel anthracycline compounds, further endeavors are needed to achieve precise chemical characterization and structural elucidation of these compounds, including NMR spectroscopy and other sophisticated analytical methods. Through this study, we hope to encourage more concerted efforts toward exploring this pristine and unexploited environment and discovering new actinobacterial species and prominent antibiotic candidates.

## Data Availability

The 16S rRNA sequences presented in this study are available in GenBank at NCBI (Accession Numbers: OQ509810–OQ509855). The whole-genome sequences of strains 378 and 549 were deposited in the NCBI GenBank under accession numbers JASKMS000000000 and JASKMT000000000, respectively.

## Supplementary Materials

The following supporting information can be downloaded at: https://www.mdpi.com/article/10.3390/microorganisms11102475/s1. Table S1: Information on sediment samples collected from four saline lakes; Table S2: Compositions of the media used in this study; Table S3: Taxonomic statistics of the isolated 255 actinobacterial strains; Table S4: Antibacterial profile of 46 bioactive strains isolated from the sediments of four saline lakes on the Northern Tibetan Plateau; Table S5: Antimicrobial activities of crude extracts from two selected *streptomyces* strains 378 and 549; Table S6: Putatively identified metabolites from liquid culture extracts of strains 378 and 549; Table S7: Biosynthesis gene clusters of secondary metabolites from strain 378 detected by anti-SMASH; Table S8: Biosynthesis gene clusters of secondary metabolites from strain 549 detected by anti-SMASH; Figure S1: Neighbor-joining phylogenetic tree based on 16S rRNA gene sequences of strain LD-9 (1509 bp) and related type species of the family *Microbacteriaceae*; Figure S2: UPLC-MS/MS spectra of putative cosmomycin compounds (**7**–**15**) from strain 378 acquired by MS^E^ method; Figure S3: UPLC-MS/MS spectra of putative kidamycin derivatives (**16**–**21**) from strain 549 acquired by MS^E^ method; Figure S4: Similar known clusters for the putative type II polyketide BGC from strain 378; Figure S5: Similar known clusters for the putative type II polyketide BGC from strain 549.
