# Supplementary material for: Bioprospecting of Actinobacterial Diversity and Antibacterial Secondary Metabolites from the Sediments of Four Saline Lakes on the Northern Tibetan Plateau
Source: Microorganisms. 2023 Oct 1;11(10):2475. doi: 10.3390/microorganisms11102475 (PMC10609225; doi:10.3390/microorganisms11102475)

Supplemental Materials

Bioprospecting of Actinobacterial Diversity and Antibacterial Secondary Metabolites from the Sediments of Four Saline Lakes on the Northern Tibetan Plateau

Shao-Wei Liu [1], Xiao-Xu Zhai [1,2,3], Di Liu [1,3], Yu-Yu Liu [1], Li-Ying Sui [4], Ke-Ke Luo [1], Qin Yang [1], Fei-Na Li [5], Arina A. Nikandrova [6,7], Arina N. Imamutdinova [6,8], Dmitrii A. Lukianov [6,8], Ilya A. Osterman [6,8], Petr V. Sergiev [6,8], Ben-Yin Zhang [9], De-Jun Zhang [9], Chun-Mei Xue [3,*] and Cheng-Hang Sun [1,2,9,*]

[1] Department of Microbial Chemistry, Beijing Key Laboratory of Antimicrobial Agents, Institute of Medicinal Biotechnology, Chinese Academy of Medical Sciences, Peking Union Medical College, Beijing 100050, China; liushaowei3535@163.com (S.-W.L.)
[2] State Key Laboratory of Plateau Ecology and Agriculture, Qinghai University, Xining 810016, China
[3] College of Life Sciences, Jiamusi University, Jiamusi 154000, China
[4] College of Marine and Environmental Sciences, Tianjin University of Science and Technology, Tianjin 300457, China
[5] Laboratory of Respiratory Diseases, Beijing Key Laboratory of Pediatric Respiratory Infection Diseases, Beijing Pediatric Research Institute, Beijing Children's Hospital, Capital Medical University, Key Laboratory of Major Diseases in Children, Ministry of Education, National Clinical Research Center for Respiratory Diseases, National Center for Children's Health, Beijing 100045, China; hbulifeina@126.com
[6] Center of Life Sciences, Skolkovo Institute of Science and Technology, 121205 Moscow, Russia
[7] Department of Biology, Lomonosov Moscow State University, 119991 Moscow, Russia
[8] Department of Chemistry, Lomonosov Moscow State University, 119991 Moscow, Russia
[9] College of Eco-Environmental Engineering, Qinghai University, Xining 810016, China; benyinzhang@qhu.edu.cn (B.-Y.Z.); djzhang@qhu.edu.cn (D.-J.Z.)
* Correspondence: jmsxcm@126.com (C.-M.X.); chenghangsun@hotmail.com (C.-H.S.)

Content

Table S1. Information of sediment samples collected from four saline lakes.
Table S2. Compositions of the media used in the study.
Table S3. Taxonomic statistics of the isolated 255 actinobacterial strains.
Table S4. Antibacterial profile of 46 bioactive strains isolated from sediments of four saline lakes on the Northern Tibetan Plateau.
Table S5. Antimicrobial activities of crude extracts from two selected *streptomyces* strains 378 and 549.
Table S6. Putatively identified metabolites from liquid culture extracts of strains 378 and 549.
Table S7. Biosynthesis gene clusters of secondary metabolites from strain 378 detected by anti-SMASH.
Table S8. Biosynthesis gene clusters of secondary metabolites from strain 549 detected by anti-SMASH.

Figure S1. Neighbor-joining phylogenetic tree based on 16S rRNA gene sequences of strain LD-9 (1509 bp) and related type species of family *Microbacteriaceae*.
Figure S2. UPLC-MS/MS spectra of putative cosmomycin compounds (**7**-**15**) from strain 378 acquired by MS^E method.
Figure S3. UPLC-MS/MS spectra of putative kidamycin derivatives (**16**-**21**) from strain 549 acquired by MS^E method.
Figure S4. Similar known clusters for the putative type II polyketide BGC from strain 378.
Figure S5. Similar known clusters for the putative type II polyketide BGC from strain 549.

Table S1. Information of sediment samples collected from four saline lakes.

Samples	Lake Name	Latitude& Longitude	Elevation (m)	pH	Salinity (g/L)
1	Dangqiong Co	31°35′24″N, 86°47′24″E	4428	8.66	110
2	Dong Co	32°7′11.99″N, 84°45′00″E	4365	8.82	40
3	Nieer Co	32°14′23.99″N, 82°13′48″E	4365	8.25	100
4	Nameless salty lake	32°32′24″N, 82°28′12″E	4366	7.66	50

Table S2. Compositions of the media used in the study.

NO.	Name	Composition (In 1.0 L distilled water)
**Used for the isolation of actinobacteria (M1-M8)**		
M1	Modified PYG medium (pH 8.0, 0 % NaCl)	Peptone 3.0 g, Yeast extract 5.0 g, Glycerol 10.0 g, Betaine 1.25 g, Sodium pyruvate 1.25 g, Agar 15.0 g, pH 8.0
M2	ISP 2 medium (pH 8.0, 0 % NaCl)	Yeast extract 4.0 g, Glucose 4.0 g, Malt extract 5.0 g, Vitamin mixture[a] 1.0 mL, Trace salt[b] 1.0 mL, Agar 15.0 g, pH 8.0
M3	Modified R2A medium (pH 8.0, 3 % NaCl)	R2A (BD) 18.6 g, NaCl 30.0 g, Agar 12.0 g, pH 8.0
M4	Modified Glycerol-arginine medium (pH 8.0, 5 % NaCl)	Arginine 2.0 g, Glycerol 12.5 g, $K_2HPO_4·3H_2O$ 2.0 g, $FeSO_4·7H_2O$ 0.01 g, $MgSO_4·7H_2O$ 0.05 g, CuSO4·$5H_2O$ 0.001 g, $ZnSO_4·7H_2O$ 0.001 g, $MnSO_4·H_2O$ 0.001 g, NaCl 50.0 g, Agar 15.0 g, pH 8.0
M5	Modified PYG medium (pH 10.0, 0 % NaCl)	Same as M1, pH 10.0
M6	ISP 2 medium (pH 10.0, 0 % NaCl)	Same as M2, pH 10.0
M7	Modified R2A medium (pH 10.0, 3 % NaCl)	Same as M3, pH 10.0
M8	Modified Glycerol-arginine medium (pH 10.0, 5 % NaCl)	Same as M4, pH 10.0
**Used for OSMAC fermentation (FM1-FM4)**		
FM1	TSB medium	TSB (BD) g, pH 8.0
FM2	ISP 2 medium	Yeast extract 4.0 g, Glucose 4.0 g, Malt extract 5.0 g, Vitamin mixture[a] 1.0 mL, Trace salt[b] 1.0 mL, pH

	8.0
FM3	Yeast extract 5.0 g, Glucose 5.0 g, Maltose 10.0 g, Cottonseed meal 10.0 g, Starch 20.0 g, pH 8.0
FM4	Starch 5.0 g, Glucose 2.0 g, Peptone 2.0 g, Yeast extract 2.0 g, Soybean meal 10.0 g, NaCl 40.0 g, K_2PO_4 0.5 g, $MgSO_4·7H_2O$ 0. 5 g, $CaCO_3$ 2.0 g, pH 8.0

Note: [a] Vitamin mixture: thiamine 0.1 g, pyridoxine 0.1 g, riboflavin 0.1g, niacin 0.1 g, biotin 0.1 g, distilled water 100.0 mL.

[b] Trace salt solution: FeSO4·7H2O 0.2 g, MnCl2·4H2O 0.01 g, ZnSO4·7H2O 0.01 g, distilled water 100.0 ml.

Table S3. Taxonomic statistics of the isolated 255 actinobacterial strains.

Taxon			No. of isolates
**Order (6)**	**Family (12)**	**Genus (21)**	
Streptomycetales	*Streptomycetaceae*	*Streptomyces*	70
Micrococcales	*Microbacteriaceae*	*Agromyces*	3
		Microbacterium	4
	Micrococcaceae	*Kocuria*	43
		Nesterenkonia	10
		Arthrobacter	27
		Citricoccus	24
		Pseudarthrobacter	10
		Paenarthrobacter	2
	Bogoriellaceae	*Georgenia*	8
		Pseudoclavibacter	2
		Salinibacterium	1
	Cellulomonadaceae	*Cellulomonas*	7
	Jonesiaceae	*Jonesia*	8
		Flavimobilis	1
Mycobacteriales	*Dietziaceae*	*Dietzia*	20
	Nocardiaceae	*Rhodococcus*	6
	Hoyosellaceae	*Hoyosella*	1
Streptosporangiales	*Nocardiopsaceae*	*Nocardiopsis*	5
Brevibacteriales	*Brevibacteriaceae*	*Brevibacterium*	1
Micromonosporales	*Micromonosporaceae*	*Micromonospora*	2
Total number of actinobacterial isolates			255

Table S4. Antibacterial profile of 46 bioactive strains isolated from sediments of four saline lakes on the Northern Tibetan Plateau. Inhibitory rates exceeding 60% are highlighted in yellow colour.

NO.	Isolates (GenBank accession NO.)	Top-hit taxon (Pairwise similarity)	Fermentation media	Inhibitory Rate (IR)											
				E. coli		*P. aeruginosa*		*K. peneumoniae*		*A. baumannii*		*S. aureus*		*E. faecalis*	
				**S**	**R**	**S**	**R**	**S**	**R**	**S**	**R**	**S**	**R**	**S**	**R**
1	104 (OQ509810)	*Streptomyces sparsus* YIM 90018^{T}, 99.9%	FM1	15.1	1.9	21.8	-5.2	7.4	22.5	34.5	26.9	-13.9	-22.0	34.1	54.3
			FM2	7.2	18.6	9.9	6.7	3.1	15.5	9.3	-16.6	7.4	-3.5	22.6	54.6
			FM3	-7.9	-0.2	30.6	-13.0	2.6	9.5	4.2	0.4	4.8	-4.7	38.9	60.4
			FM4	-8.6	0.4	-3.7	-6.8	6.5	-1.6	-9.7	-4.6	-5.5	-9.2	20.0	4.8
2	116 (OQ509811)	*Streptomyces sparsus* YIM 90018^{T}, 99.7%	FM1	14.4	0.0	7.6	0.7	-11.7	17.7	6.6	62.8	2.4	-2.3	21.1	38.8
			FM2	11.2	6.6	-17.1	-0.4	0.3	17.8	20.5	-8.8	-9.0	-6.5	11.0	47.0
			FM3	-12.8	-2.9	-6.3	-3.6	-2.6	-10.7	-1.8	-1.6	-8.0	-7.6	24.5	46.8
			FM4	-13.5	-6.8	-5.6	-2.8	-0.5	-7.4	-3.9	-1.7	-14.1	-9.7	30.5	19.9
3	S683 (OQ509812)	*Streptomyces rishiriensis* NBRC 13407^{T}, 98.3%	FM1	27.8	-17.3	0.9	-2.4	56.3	47.5	25.1	-5.3	79.7	50.6	67.1	24.4
			FM2	-5.8	-4.5	-23.3	-11.0	4.3	25.3	-16.8	-9.5	30.1	49.2	44.4	-3.1
			FM3	-5.7	-7.0	10.6	-33.5	-8.4	-8.9	7.0	-8.6	27.0	42.7	75.6	15.6
			FM4	4.0	-10.2	-11.1	-11.7	-4.6	-2.8	-5.9	-8.1	-9.7	10.5	12.2	-5.3
4	S503 (OQ509813)	*Streptomyces sparsus* YIM 90018^{T}, 99.7%	FM1	0.0	-8.4	-7.7	-17.6	-0.5	-2.3	0.6	-11.9	9.6	-15.4	0.9	-18.2
			FM2	-3.7	-10.5	-14.9	-16.0	1.2	-17.8	-3.3	-15.6	18.5	4.5	-19.6	5.8
			FM3	-19.4	-11.4	-17.0	-23.1	0.0	-7.0	-14.4	5.6	-14.6	8.5	3.1	-6.4
			FM4	1.2	-8.9	1.1	-3.5	0.3	-17.6	1.7	9.7	1.1	62.0	-17.3	-13.6
5	S600 (OQ509814)	*Streptomyces sparsus* YIM 90018^{T}, 99.6%	FM1	6.9	-9.9	2.0	-8.1	-24.3	-2.1	7.0	-7.7	-9.4	-2.0	-1.3	-0.7
			FM2	-11.9	-10.5	-10.4	-10.2	-15.4	-4.0	88.8	-8.8	-5.2	-1.8	-7.8	-1.8

			FM3	-7.0	-4.9	-12.2	-13.5	-11.1	-13.3	-17.3	-15.3	-9.2	-2.3	-4.4	-7.8
			FM4	-8.4	16.0	-10.6	-15.2	-18.3	-10.2	-12.5	-18.3	-10.9	-5.0	-1.8	3.8
6	S542 (OQ509815)	*Streptomyces sparsus* YIM 90018^{T}, 99.7%	FM1	-11.8	-23.4	-1.5	-9.0	-18.1	-7.1	71.4	28.0	-8.2	-8.2	7.8	-10.8
			FM2	-10.1	-8.5	-8.0	-12.5	-12.2	-1.6	-8.3	-9.2	-7.5	-2.8	-9.0	0.2
			FM3	-4.2	-3.0	-11.0	10.3	-3.5	-6.2	11.8	-6.5	6.4	14.2	7.6	-6.8
			FM4	-16.4	-0.7	-9.4	-3.5	-9.4	-7.6	-8.4	-3.7	12.3	-1.2	-4.5	21.3
7	S549 (OQ509816)	*Streptomyces sparsus* YIM 90018^{T}, 99.9%	FM1	9.3	-9.2	-13.2	-8.1	0.6	-3.6	-0.5	-1.9	-5.7	-3.3	-12.6	-0.6
			FM2	-6.3	-7.3	-1.0	-13.3	-7.2	-6.6	1.2	10.1	-16.5	6.7	-11.2	-2.9
			FM3	-16.4	-0.6	4.2	-6.8	-11.8	-6.7	-14.7	-1.9	46.0	-0.3	-2.1	23.2
			FM4	78	85.1	11.1	-13.8	-12.3	-15.2	-11.5	-13.0	91.7	81.7	86.1	76.7
8	S502 (OQ5098117)	*Streptomyces sparsus* YIM 90018^{T}, 99.7%	FM1	1.0	-0.2	-3.9	15.7	-8.9	-7.2	-7.4	21.8	5.7	-0.9	-7.8	-6.8
			FM2	-5.9	-9.4	-11.5	-2.1	-2.1	-3.3	-7.7	-2.1	-3.6	-1.2	-6.8	-2.5
			FM3	-13.7	-6.5	6.3	8.4	-10.0	-0.5	-4.3	-10.0	8.0	1.7	4.5	-9.5
			FM4	-6.2	-6.7	7.0	0.2	-3.8	-6.4	-9.0	-2.9	64.1	78.4	42.6	71.2
9	378 (OQ509818)	*Streptomyces marokkonensis* Ap1^{T}, 99.4%	FM1	-8.3	-4.7	-0.2	-8.6	-5.1	-8.8	-3.1	-10.8	8.5	-8.9	-9.5	-10.9
			FM2	-9.2	-7.5	-4.1	-9.4	-9.4	13.5	-11.3	-4.1	3.1	3.1	-2.1	-10.0
			FM3	-6.4	-7.3	-13.0	-7.4	-15.1	-10.3	-18.1	-4.1	15.4	8.9	1.0	-7.1
			FM4	83.5	-3.5	-13.4	-8.9	15.2	-4.3	-8.5	3.4	76.6	78.7	84.4	78.0
10	267 (OQ5098119)	*Streptomyces bacillaris* NBRC 13487^{T}, 99.2%	FM1	-5.5	-0.7	-7.8	1.1	-8.3	-7.8	-13.9	-1.2	14.1	0.8	27.9	40.2
			FM2	-9.6	27.2	-8.8	-1.3	-6.7	-4.3	-3.9	8.8	6.7	15.6	32.2	13.3
			FM3	-5.7	-13.3	-12.0	-1.3	-4.2	-6.2	72.2	-7.7	1.5	7.8	-4.4	30.4
			FM4	-6.5	-6.0	-13.8	-10.0	-1.8	-7.9	-2.7	-9.3	27.0	29.2	-6.8	-8.2
11	X132 (OQ509820)	*Streptomyces rishiriensis* NBRC 13407^{T}, 98.8%	FM1	-14.9	-4.3	96.3	-5.4	-7.4	-9.5	-1.2	-21.8	-7.0	-11.6	6.5	-1.6
			FM2	12.6	-13.3	8.2	24.1	-15.0	-10.1	51.5	-8.8	-9.2	-10.3	-4.4	21.1
			FM3	-6.6	-9.4	-0.7	6.8	-11.7	-10.8	25.1	-7.6	-4.0	-19.7	7.5	8.0

			FM4	6.6	-1.6	10.9	-12.1	-11.6	-10.4	35.7	-8.1	-7.1	7.2	-1.2	22.5
12	X93-1 (OQ509821)	*Streptomyces marokkonensis* Ap1^T, 99.1%	FM1	11.4	-1.5	-9.0	15.0	5.5	-11.3	-17.7	-10.4	-4.3	-3.8	7.8	-5.6
			FM2	4.6	14.0	-2.7	5.2	2.4	-1.8	-16.3	-11.4	-4.4	-1.1	10.6	-5.7
			FM3	11.1	-15.0	-1.9	-4.5	-7.4	-3.6	12.5	-17.8	-12.9	-15.2	-2.0	-3.2
			FM4	4.3	-8.8	8.6	-3.7	14.5	-1.1	-9.2	-1.8	66.8	67.6	13.5	-12.6
13	X326 (OQ509822)	*Streptomyces sparsus* YIM 90018^T, 99.9%	FM1	-4.9	-14.3	96.3	9.2	9.5	-0.7	-10.2	-7.2	-6.6	5.5	-13.3	-5.7
			FM2	-8.6	-13.3	8.2	4.1	6.4	-10.1	-6.2	-41.8	-10.7	2.4	17.2	12.9
			FM3	-7.7	-9.4	-0.7	8.6	-16.5	-15.5	10.4	-52.3	-5.2	-7.4	14.1	-10.7
			FM4	-7.7	-1.6	10.9	-11.6	-14.8	-12.6	-3.6	-24.1	-2.9	14.5	-1.0	10.2
14	X15-2 (OQ509823)	*Streptomyces sparsus* YIM 90018^T, 99.9%	FM1	40.3	-11.7	-15.0	7.2	3.2	61.8	13.8	-14.5	-23.9	6.4	-10.3	-7.7
			FM2	-6.3	-12.5	-16.1	2.6	18.5	15.4	-9.2	-6.0	-7.0	36.8	-11.6	-5.2
			FM3	-17.7	-8.3	2.2	1.2	11.3	7.1	12.5	-6.4	-3.4	11.5	-14.0	-9.8
			FM4	-4.0	-10.7	-7.5	-9.3	-0.2	2.2	28.5	-9.9	-7.8	2.8	-18.4	-8.8
15	99-1 (OQ509824)	*Arthrobacter pascens* DSM 20545^T, 99.9%	FM1	-7.1	-5.7	-8.1	17.1	-12.2	-11.0	74.1	-8.7	-5.5	-15.3	2.1	-1.8
			FM2	-1.8	-9.9	-6.8	11.4	-4.8	-16.4	11.4	-7.4	-3.8	-7.2	-7.1	9.5
			FM3	-7.7	-3.0	-5.3	24.2	-12.5	-0.8	64.9	-5.3	-3.5	-9.1	-17.1	-7.0
			FM4	-7.7	-6.3	-3.8	3.8	-16.1	-1.4	9.8	-4.2	-5.7	2.9	-31.2	-6.5
16	60 (OQ5098205)	*Arthrobacter oryzae* KV-651^T, 99.9%	FM1	9.5	-8.2	-10.1	2.2	7.2	18.0	75.5	16.5	-8.2	-6.7	-12.5	36.2
			FM2	16.1	-2.7	-11.9	-2.6	20.1	3.5	19.9	43.9	21.5	-16.8	18.9	37.2
			FM3	8.3	0.0	-11.3	-11.8	22.1	-7.7	6.8	22.0	-15.2	-2.9	-12.9	36.8
			FM4	2.5	10.6	14.6	-17.8	4.5	-17.7	-6.7	-8.5	-1.4	3.4	25.0	35.7
17	S525 (OQ509826)	*Arthrobacter halodurans* JSM 078085^T, 99.7%	FM1	0.3	-12.6	-12.0	2.6	-10.4	-11.5	-5.4	-11.1	-5.2	-10.0	2.8	2.3
			FM2	1.0	-14.8	-21.9	-4.8	-11.3	-6.6	-10.9	-1.1	21.1	-8.4	-10.6	-11.4
			FM3	-9.5	-9.4	12.7	-2.2	-15.9	-4.5	-3.7	-9.8	24.9	7.0	33.9	48.7
			FM4	75.3	-15.9	-13.0	-11.7	-6.0	-6.2	-6.5	-18.9	39.3	62.2	24.0	44.5

18	S706	*Arthrobacter oryzae* KV-651^T,	FM1	-8.7	-8.4	-11.6	-8.9	1.0	-8.9	11.4	4.1	-1.5	10.3	15.2	-13.1
	(OQ509827)	100.0%	FM2	-0.5	-9.2	-8.9	-8.6	-8.6	-4.6	-4.4	2.2	-1.0	0.8	-1.2	-7.1
			FM3	12.3	-18.9	-1.1	-14.4	-10.4	3.7	-18.9	17.3	36.5	1.9	-2.4	1.6
			FM4	-8.2	-5.3	-9.3	-3.2	-12.7	-6.9	-2.8	-9.7	31.5	67.6	26.5	63.1
19	216	*Arthrobacter oryzae* KV-651^T,	FM1	-2.7	-15.2	-4.7	-5.7	0.7	14.7	-12.2	-9.6	24.9	-2.8	-9.2	-1.6
	(OQ509828)	100.0%	FM2	-10.1	-6.1	-6.1	-8.6	-1.5	77.5	-6.3	-5.6	11.3	6.1	-4.8	-4.2
			FM3	-9.8	-4.1	-7.9	-9.8	-1.8	11.2	-6.3	-8.1	19.3	2.8	-5.4	-0.4
			FM4	-7.2	-13.2	-5.2	-11.5	-19.1	14.2	-18.1	-6.8	7.7	-6.7	-6.6	-4.0
20	X76	*Arthrobacter halodurans* JSM	FM1	-13.4	-6.9	-3.3	8.8	-1.7	6.1	3.6	-1.5	-8.6	77.5	4.8	-3.3
	(OQ509829)	078085^T, 99.9%	FM2	-11.8	-3.3	-2.4	-0.7	3.3	15.6	-6.2	-1.8	-11.2	-1.9	56.3	47.5
			FM3	-4.9	-3.4	-1.5	-0.5	-17.7	-13.1	3.0	-9.1	-4.2	-6.4	4.3	25.3
			FM4	-1.7	-8.5	-8.0	-2.8	-10.6	-10.7	-16.8	-5.1	-8.8	-1.1	-8.4	-8.9
21	X2	*Arthrobacter oryzae* KV-651^T,	FM1	-17.1	-13.0	-11.0	9.6	-5.3	4.2	-4.8	-9.4	-10.5	83.5	-4.6	2.8
	(OQ509830)	100.0%	FM2	-1.8	-0.7	-9.4	11.6	-2.4	14.9	-3.4	-15.1	-10.3	-5.1	-14.1	20.4
			FM3	-7.7	-12.7	-9.6	4.9	-8.4	3.3	-0.4	15.2	-4.3	-2.4	-2.3	3.8
			FM4	-17.7	-10.8	-13.4	5.5	-5.3	-11.4	-6.4	-7.9	-3.1	-3.9	-6.7	16.7
22	X23-3	*Arthrobacter oryzae* KV-651^T,	FM1	-7.3	-6.0	9.2	-1.1	6.4	7.3	12.5	-13.2	-3.1	-3.4	-3.2	-5.7
	(OQ509831)	99.9%	FM2	-5.0	11.6	21.8	6.3	36.8	21.1	-17.8	4.0	-5.6	6.2	-11.3	-5.4
			FM3	-16.0	-14.4	0.0	-8.5	11.5	9.0	95.2	15.2	-9.2	-2.6	-10.9	-0.8
			FM4	-8.7	-4.2	-9.2	-8.3	2.8	-10.9	-7.2	-8.3	-7.8	-23.2	-9.5	-6.3
23	23-2	*Kocuria rosea* DSM 20447^T,	FM1	10.2	-0.6	16.5	26.1	37.3	88.8	60.1	86.2	-9.2	-3.0	22.3	27.0
	(OQ509832)	99.0%	FM2	-7.9	-13.4	-8.0	0.6	19.9	59.1	17.3	82.6	-0.3	-5.2	16.7	5.1
			FM3	3.3	-1.4	-11.0	-31.9	18.4	49.6	20.6	80.3	-12.5	-2.9	22.3	23.3
			FM4	-2.8	14.0	2.0	-12.7	13.5	34.2	-17.0	41.1	-2.8	-3.4	7.8	12.9
24	185	*Kocuria Polaris* CMS 76or^T,	FM1	7.4	-1.2	30.9	10.7	-4.4	21.1	19.7	20.7	25.7	-13.4	25.1	41.7

	(OQ509833)	99.9%	FM2	8.6	14.0	12.5	8.9	7.5	8.0	1.2	-5.7	-5.8	-13.2	20.9	60.1
			FM3	1.2	23.6	20.6	9.2	-1.2	22.5	-1.4	-6.4	48.8	6.3	39.2	43.9
			FM4	-5.1	-6.6	-5.6	-3.8	-8.1	-11.3	-5.0	-4.7	15.6	-7.6	43.7	56.1
25	192 (OQ509834)	*Kocuria Polaris* CMS 76or^T, 99.9%	FM1	-5.0	-8.8	15.3	3.7	30.4	45.3	92.7	53.2	-8.2	-59.6	-1.0	17.0
			FM2	-8.4	-9.6	2.2	-15.9	-0.6	21.8	92.2	53.6	-0.7	-2.0	-8.2	17.1
			FM3	-13.4	-9.6	-3.9	-5.4	7.7	15.7	94.5	18.4	9.9	-2.0	16.0	32.3
			FM4	-13.3	6.5	-10.1	-7.8	7.8	-5.6	91.3	-5.6	3.6	-2.2	3.3	35.5
26	S936 (OQ509835)	*Kocuria Polaris* CMS 76or^T, 98.9%	FM1	-11.6	-16.0	0.0	-21.6	-10.1	-0.8	5.9	16.3	-8.2	-4.3	12.3	-3.5
			FM2	-13.4	-8.1	96.0	-8.9	-12.6	-8.3	-5.4	8.8	8.0	-9.5	-3.8	-3.6
			FM3	-11.0	-3.4	-2.9	-1.1	-0.1	-4.1	-18.8	-4.8	22.1	-11.8	10.0	-6.4
			FM4	-9.7	-13.3	-2.5	-9.3	-2.7	-10.1	-12.9	-2.3	54.6	75.6	41.3	70.8
27	S676 (OQ509836)	*Kocuria Polaris* CMS 76or^T, 99.9%	FM1	-18.6	-16.1	-6.3	-16.1	-5.1	-1.7	-9.9	4.9	-6.7	-0.6	-0.8	-7.1
			FM2	-16.1	-15.6	-7.8	-8.2	-6.8	-2.9	9.1	-1.7	25.7	-2.6	-2.1	-12.0
			FM3	-14.9	-11.5	-9.0	3.3	-13.7	-0.5	-15.9	-4.5	9.8	0.3	32.0	6.2
			FM4	11.6	14.0	-12.7	-5.0	-1.2	-9.7	15.6	5.2	40.4	69.9	45.3	-1.2
28	S629 (OQ509837)	*Kocuria dechangensis* NEAU-ST5-33^T, 99.0%	FM1	-6.3	1.6	-9.2	-5.4	7.9	-3.4	-3.5	-0.7	14.1	-9.5	4.5	-6.2
			FM2	-14.1	-29.8	-12.2	-7.3	29.1	-5.5	-1.2	-4.8	-4.4	-3.6	1.7	-8.7
			FM3	22.2	-9.3	-15.9	-18.0	28.7	-7.3	-8.9	-1.1	-15.4	10.6	1.2	-8.9
			FM4	-10.2	-1.4	-15.4	-5.0	39.9	-4.7	-1.4	-8.8	49.6	71.5	7.1	58.2
29	S573 (OQ509838)	*Kocuria dechangensis* NEAU-ST5-33^T, 99.0%	FM1	2.7	6.1	-5.5	-15.2	-15.6	-6.9	8.0	1.1	-2.6	-6.7	9.5	7.3
			FM2	10.1	4.2	-14.3	-9.9	-22.5	-16.7	-11.0	-8.9	12.3	-4.5	0.0	21.1
			FM3	9.8	9.4	-13.4	-10.6	-20.9	-13.5	-4.0	-5.0	9.0	-8.6	-1.2	15.6
			FM4	7.2	9.4	-1.9	-12.0	-15.9	-8.2	-4.6	-7.7	49.6	67.6	33.4	70.2
30	S910 (OQ509839)	*Nesterenkonia halotolerans* YIM 70084^T, 99.9%	FM1	-8.3	0.6	-5.3	-7.6	-8.3	-9.3	9.0	-5.2	13.1	-2.5	3.5	-15.6
			FM2	-9.2	3.5	-16.3	-9.9	-11.6	-7.1	-15.2	-9.6	-6.5	-4.2	-13.5	-18.0

			FM3	-6.4	3.6	-17.5	-8.1	-17.4	-3.1	-17.3	-8.0	-6.4	-1.7	-3.8	-11.3
			FM4	-12.7	0.1	-11.8	-13.6	-9.8	-1.1	-12.0	-11.7	59.3	73.0	46.7	71.1
31	LD-11 (OQ509840)	*Nesterenkonia halotolerans* YIM 70084^T, 99.9%	FM1	5.3	-5.8	-9.4	5.7	-3.1	-7.3	-4.1	8.3	1.3	-8.1	7.4	9.7
			FM2	-9.2	-4.9	-3.5	-6.3	-2.5	-8.2	-12.9	-3.3	-8.5	-9.3	-7.6	2.1
			FM3	-7.0	-1.7	-15.0	3.4	-2.9	-7.4	-6.2	-6.1	-1.1	-7.0	3.8	30.8
			FM4	-7.1	2.5	-16.1	-13.4	-9.7	-6.9	-3.2	-7.7	29.8	65.0	34.6	41.6
32	S491 (OQ509841)	*Nesterenkonia lutea* YIM 70081^T, 99.3%	FM1	4.5	-9.9	-11.2	-9.8	-1.8	-12.3	-13.5	-9.8	-5.7	-5.6	-6.4	-6.6
			FM2	-5.5	-15.9	-15.6	-6.2	-5.4	-6.6	-16.6	-2.8	-6.5	-17.0	-20.1	-9.1
			FM3	-17.2	-12.9	1.1	-6.2	-4.9	1.4	-6.7	-3.6	46.0	-13.4	-4.5	-0.8
			FM4	4.3	7.1	-8.3	-1.3	-2.0	-7.0	-7.4	-12.9	62.0	14.5	13.5	23.0
33	244-1 (OQ509842)	*Nesterenkonia lutea* YIM 70081^T, 99.4%	FM1	-7.8	-6.7	-10.7	-5.8	-2.5	-21.0	-13.2	32.8	14.7	8.4	15.9	-12.2
			FM2	-12.3	1.7	-15.7	-12.7	-14.5	-23.9	-6.8	16.3	10.3	12.0	47.2	-15.6
			FM3	2.0	-6.0	-14.8	-11.0	-6.0	-7.0	-21.2	-12.8	-14.7	-7.0	18.2	5.3
			FM4	-14.6	6.7	-11.6	-9.9	-6.4	-3.4	-14.3	-14.7	56.8	57.1	23.5	61.6
34	46 (OQ509843)	*Dietzia psychralcaliphila* JCM 10987^T, 99.7%	FM1	17.4	0.2	14.5	-7.7	17.6	42.4	-0.2	64.7	-9.3	-4.1	13.5	44.3
			FM2	-8.1	-12.1	-8.8	-14.7	4.4	26.1	38.5	-8.8	-10.1	-2.6	8.2	14.2
			FM3	-12.8	-14.1	2.4	-10.2	4.2	15.1	22.9	4.1	-7.6	-2.3	24.3	31.0
			FM4	-4.4	7.6	-16.2	-12.3	-3.1	37.7	4.8	-1.7	-6.9	-6.3	22.5	19.7
35	S679 (OQ509844)	*Dietzia psychralcaliphila* CMS 76or^T, 99.9%	FM1	-10.4	-8.3	2.2	96.3	-7.9	6.0	2.9	14.7	-6.4	-12.1	-6.9	17.4
			FM2	-9.4	-10.7	-7.5	8.2	-5.9	-8.8	-4.0	3.5	-6.8	-10.5	20.8	-9.7
			FM3	-8.0	-14.3	-11.5	-0.7	-9.6	-9.2	-6.8	-2.9	-7.9	-14.9	11.6	-0.4
			FM4	-13.1	7.1	-8.9	10.9	-5.3	-6.3	-3.1	-7.7	15.2	44.6	17.0	13.9
36	298 (OQ509845)	*Dietzia psychralcaliphila* JCM 10987^T, 99.2%	FM1	-1.7	-7.8	-8.1	-3.7	-12.0	-10.6	-8.8	-11.9	15.7	-8.1	-12.8	-9.3
			FM2	-9.6	-1.1	-13.3	-1.3	-13.2	-13.1	-10.9	-6.1	24.2	11.4	-4.0	-7.1
			FM3	-0.5	-9.7	-6.8	-4.6	-4.0	-15.6	-11.2	-13.7	48.1	-0.8	-16.4	-5.3

			FM4	-6.4	-7.2	-13.8	-5.1	95.2	-9.2	-12.0	-4.8	60.4	14.7	38.1	45.6
37	S631	*Pseudarthrobacter*	FM1	-7.8	-10.8	-5.6	-5.3	-0.3	-5.8	-7.3	5.6	9.5	-13.4	28.0	-16.2
	(OQ509846)	*sulfonivorans* ALL^{T}, 99.6%	FM2	-2.3	-12.0	-11.3	-15.0	-15.5	-12.9	-2.3	-8.5	12.3	16.2	5.9	-14.7
			FM3	2.0	-13.2	15.6	-13.8	-12.4	-10.8	-6.7	-9.1	18.0	-0.8	31.1	40.7
			FM4	-14.6	1.7	-8.0	-9.8	-11.4	-10.3	-2.0	-8.9	75.8	61.6	19.2	70.7
38	S511	*Pseudarthrobacter*	FM1	-6.6	-4.3	96.3	-7.1	-0.9	4.1	13.5	8.4	16.2	28.4	10.7	20.7
	(OQ509847)	*sulfonivorans* ALL^{T}, 99.6%	FM2	-7.0	-3.3	8.2	-12.7	-9.1	9.0	-17.3	11.3	9.0	-17.0	-12.1	3.1
			FM3	-3.0	-9.4	-0.7	-6.7	-8.5	-0.3	20.0	12.5	37.3	-19.8	10.6	-2.2
			FM4	-8.9	-11.6	10.9	-3.5	-8.9	-8.4	22.2	5.1	53.5	45.1	26.5	59.3
39	91	*Nocardiopsis yanglingensis*	FM1	78.6	0.2	13.4	-4.4	17.2	12.9	57.8	23.1	-8.9	-1.2	-1.2	2.0
	(OQ509848)	A18^{T}, 98.7%	FM2	-0.8	-2.0	9.7	2.4	14.1	-10.7	41.1	-13.9	-9.2	-5.0	7.6	22.5
			FM3	-9.0	-7.3	19.2	-27.2	-1.0	10.2	8.7	-12.7	-9.0	-2.0	24.8	34.9
			FM4	-8.4	-9.1	-5.1	-3.4	10.3	-8.0	-8.6	-3.8	-1.1	-1.2	13.5	18.4
40	85	*Nocardiopsis flavescens*	FM1	0.5	14.0	-3.3	-1.8	-10.5	-9.7	10.4	-5.6	-7.9	-6.0	9.6	17.8
	(OQ509849)	CGMCC 4.5723^{T}, 99.2%	FM2	9.5	-12.2	-14.5	-3.9	-4.7	-9.3	-13.4	-7.7	79.9	83.4	84.2	10.7
			FM3	0.5	-1.1	-16.8	-16.0	-10.5	-3.4	-9.8	-2.8	-7.7	-6.9	42.5	37.1
			FM4	-1.2	-0.5	-7.2	-15.3	-2.6	-3.3	-5.2	-4.2	-4.8	-5.4	32.9	44.3
41	LD-10	*Georgenia soli* DSM 21838^{T},	FM1	-11.7	5.7	-8.1	-16.8	-5.7	-4.6	2.3	10.8	4.9	-10.9	12.1	9.3
	(OQ509850)	98.7%	FM2	-13.6	9.9	-6.8	-16.8	-4.9	-7.5	-13.5	-18.9	12.3	-4.7	-4.7	13.1
			FM3	0.5	3.0	-5.3	-11.4	5.8	-11.6	-12.4	-5.2	4.1	14.8	0.7	0.4
			FM4	6.4	6.3	-3.8	-3.3	8.9	-8.9	-10.7	-1.1	53.8	67.6	29.9	66.4
42	S532	*Cellulomonas oligotrophica*	FM1	4.2	-5.0	-4.9	-7.8	-4.1	-2.9	-2.1	-7.5	2.8	4.0	4.4	-5.5
	(OQ509851)	Kc5^{T}, 99.7%	FM2	9.4	-8.2	-14.1	-18.8	-8.2	-8.2	-0.3	-2.3	36.2	16.2	0.2	-8.5
			FM3	9.9	-8.1	-18.3	-12.0	-5.3	-1.6	-0.7	-14.2	-12.9	19.8	19.4	-7.5
			FM4	0.8	-17.4	-12.0	-13.8	-9.3	-8.6	-7.2	-15.7	37.6	60.9	15.9	-10.9

43	S675 (OQ509852)	*Jonesia quinghaiensis* DSM 15701^T, 99.9%	FM1	2.3	-11.5	-3.3	0.1	-8.3	-6.8	0.8	7.1	-15.2	-6.2	-0.3	44.5
			FM2	-10.5	-8.1	-5.3	-13.5	-4.7	-2.6	-2.9	-7.7	9.5	-14.5	-12.6	10.4
			FM3	-12.2	-5.4	-9.7	4.6	-7.3	-5.6	-9.5	-10.3	23.7	11.7	24.4	17.0
			FM4	-15.7	-6.8	-11.0	-3.1	-6.3	-5.1	-6.7	-14.5	42.1	75.6	56.4	26.1
44	453-2 (OQ509853)	*Micromonospora olivasterospora* DSM 43868^T, 99.5%	FM1	-3.5	16.5	-5.1	-15.0	15.7	9.5	4.0	-10.3	-5.2	-9.8	8.2	64.1
			FM2	-8.1	8.0	-5.9	-13.2	8.4	18.5	-7.9	-14.0	-5.1	-6.7	-18.7	16.4
			FM3	-4.4	1.0	-13.3	-14.5	10.1	-13.1	-6.4	-14.7	-9.8	-1.1	48.1	34.6
			FM4	17.8	2.0	-10.9	-9.3	2.1	-4.8	-9.8	-11.3	-3.6	1.6	21.6	50.0
45	LD-7 (OQ509854)	*Hoyosella rhizosphaerae* J12GA03^T, 100.0%	FM1	-11.1	-9.5	3.0	9.2	-18.0	-4.4	-12.6	4.0	-9.8	-8.9	8.8	-7.0
			FM2	-15.4	-3.7	0.4	0.5	-5.8	-8.3	-16.0	2.2	-2.6	-3.5	16.1	-9.3
			FM3	-11.8	-8.4	8.0	-2.7	-3.0	-2.6	-11.0	-1.8	-14.2	-4.8	18.0	-7.5
			FM4	-16.1	-8.1	6.3	4.3	-4.1	-8.8	-16.5	-9.8	56.8	67.4	38.8	67.3
46	S697 (OQ509855)	*Agromyces indicus* NIO-1018^T, 99.0%	FM1	-18.6	2.1	2.6	-4.4	-9.9	-7.8	-16.3	-7.2	9.3	2.8	11.1	2.7
			FM2	-9.3	-0.6	-4.8	-6.9	-5.6	-0.9	-13.5	-0.9	28.8	11.1	5.2	5.1
			FM3	-14.4	-2.7	-2.2	5.4	-6.3	-4.7	2.7	12.2	17.5	14.8	16.1	6.7
			FM4	-6.5	-9.9	-11.7	-11.4	-4.4	-9.4	-4.4	-12.9	47.0	33.1	35.6	60.0

Table S5. Antimicrobial activities of crude extracts from two selected *Streptomyces* strains 378 and 549. S, sensitive; R, resistant.

**Isolates**	**Fermentation media**	**Inhibitory Rate (IR)**											
		E. coli		*P. aeruginosa*		*K. peneumoniae*		*A. baumannii*		*S. aureus*		*E. faecalis*	
		**S**	**R**	**S**	**R**	**S**	**R**	**S**	**R**	**S**	**R**	**S**	**R**
**Inhibitory zone (diameter, cm), tested by the paper-disk diffusion method with extracts of 100 mL medium**													
378	FM4	0.9								1.5	1.6	1.0	1.4
549	FM4	1.1	1.0					1.0	0.8	1.1	1.4	1.1	1.3
**Inhibitory rate (IR, %), tested by the turbidometric assay (OD_{600}) with extracts of 2 mL medium**													
378	FM4	83.5								76.6	78.7	84.4	78.0
549	FM4	78	85.1							91.7	81.7	86.1	76.7

Table S6. Putatively identified metabolites from liquid culture extracts of strains 378 and 549. t_R, retention time in UPLC.

No.	Strain	t_R (min)	Observed m/z	Calculated m/z	Putative chemical	Molecular formula	Chemical structure
1	378	4.13	1189.5858	1189.5829	cosmomycin D	$C_{60}H_{88}N_2O_{22}$	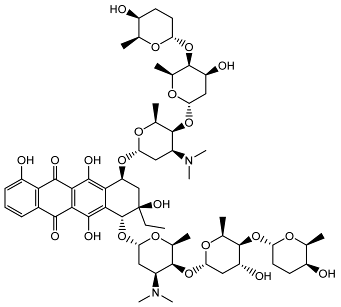
2	378	4.74	1173.5915	1173.5880	cosmomycin C	$C_{60}H_{88}N_2O_{21}$	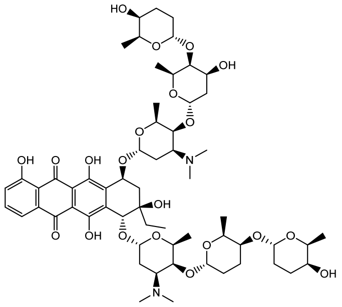
3	378	7.81	772.3502	772.3466	cosmomycin B	$C_{40}H_{53}NO_{14}$	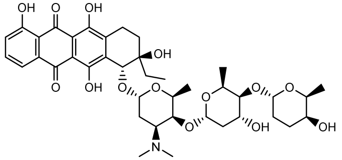

4	378	8.27	756.3540	756.3517	cosmomycin A	$C_{40}H_{53}NO_{13}$	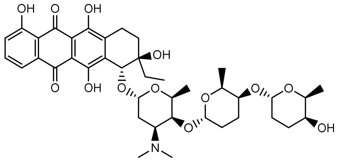
5	549	5.17	747.3440	747.3415	hedamycin	$C_{41}H_{50}N_2O_{11}$	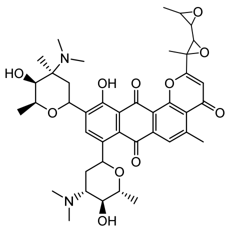
6	549	6.70	689.3432	689.3460	kidamycin	$C_{39}H_{48}N_2O_9$	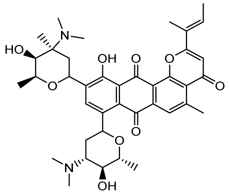
7	378	4.38	1187.5710	1187.5672	dehydrocosmomycin D	$C_{60}H_{86}N_2O_{22}$	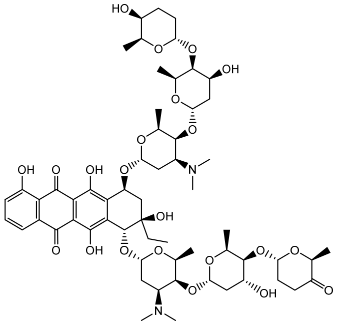

8	378	4.96	1171.5734	1171.5723	dehydrocosmomycin C	$C_{60}H_{86}N_2O_{21}$	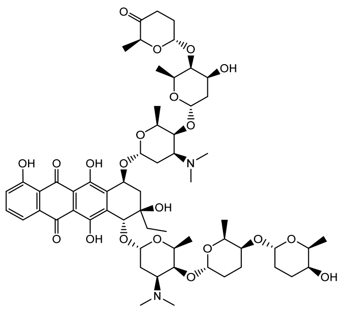
9	378	5.16	1171.5734	1171.5723	A447 D	$C_{60}H_{86}N_2O_{21}$	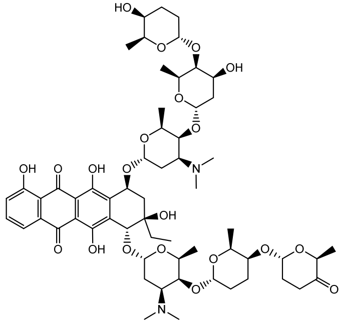
10	378	5.73	1157.5942	1157.5930	A447 C	$C_{60}H_{88}N_2O_{20}$	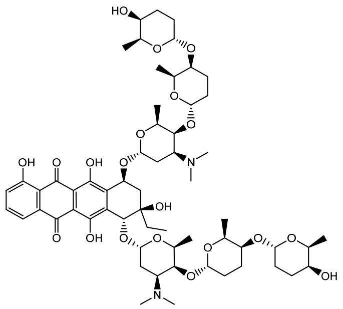

11	378	6.83	1075.5158	1075.5148	*β*-rhodomycin S-4	$C_{54}H_{78}N_2O_{20}$	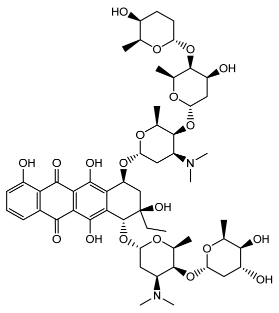
12	378	4.83	1173.5925	1173.5880	unknown	$C_{60}H_{88}N_2O_{21}$	CosD lacks one hydroxyl group in the aglycone
13	378	6.21	1155.5762	1155.5774	unknown	$C_{60}H_{86}N_2O_{20}$	A447 C contains a double bond in one outermost sugar (rho)
14	378	8.09	754.3381	754.3360	cosmocarcin C	$C_{40}H_{51}NO_{13}$	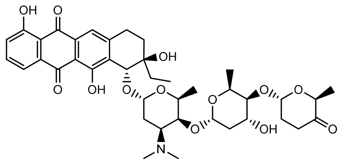
15	378	8.85	740.3557	740.3568	unknown	$C_{40}H_{51}NO_{12}$	dehydroxylated CosA
16	549	9.48	705.3298	705.3309	epoxykidamycin	$C_{39}H_{48}N_2O_{10}$	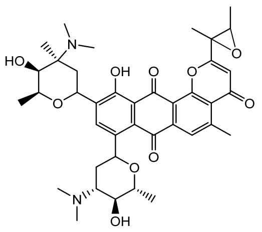

17	549	7.64	687.3245	687.3203	dehydrokidamycin	$C_{39}H_{46}N_2O_9$	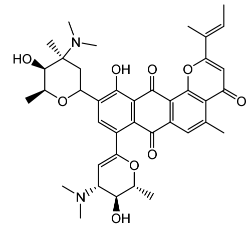
18	549	9.21	717.3650	717.3673	saptomycin C1	$C_{41}H_{52}N_2O_9$	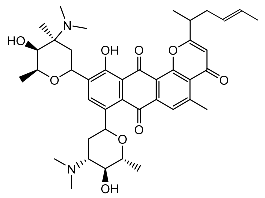
19	549	7.96	719.3841	719.3829	dihydrosaptomycin C1	$C_{41}H_{54}N_2O_9$	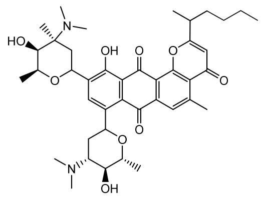
20	549	6.26	731.3477	731.3465	neopluramycin (acetylkidamycin)	$C_{41}H_{50}N_2O_{10}$	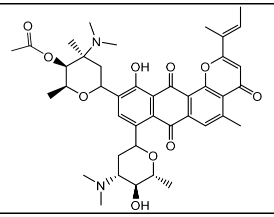
21	549	6.13	733.3632	733.3622	rubiflaoin E	$C_{41}H_{52}N_2O_{10}$	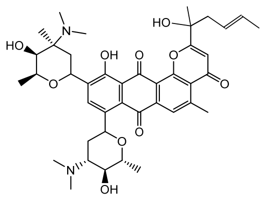

Table S7. Biosynthesis gene clusters of secondary metabolites from strain 378 detected by anti-SMASH.

	Type	Location	Most similar known cluster	Similarity
1	T2PKS, oligosaccharide	50,817-123,326	cytorhodin	84%
2	T3PKS	382,152-423,204	alkylresorcinol	100%
3	butyrolactone	567,949-579,331	coelimycin P1	8%
4	RiPP-like, NRP-metallophore, NRPS	296,611-365,956	coelichelin	100%
5	Terpenehopene	437,769-464,456	hopene	92%
6	Melanin	337,853-348,215	melanin	57%
7	RRE-containing	272,299-292,586	belactosin A/belactosin C	12%
8	T1PKS,NRPS	19,354-70,069	rifamorpholine	9%
9	Terpene	275,236-297,056	streptozotocin	23%
10	RiPP-like	19,951-30,172	BD-12	10%
11	Terpene	16,385-38,550	geosmin	100%
12	T1PKS, butyrolactone	235,739-283,174	tambjamine BE-18591	17%
13	NRPS	193,312-271,916	cyclofaulknamycin	8%
14	Lanthipeptide-class-i	191,215-217,668		
15	Terpene	347-21,005	isorenieratene	100%
16	Terpene	152,076-172,999	albaflavenone	100%
17	NI-siderophore	7,443-20,585		
18	T1PKS	160,742-193,114	s56-p1	11%
19	Ectoine	93,981-104,379	ectoine	100%
20	T1PKS	3,838-47,806	chlorothricin/deschlorothricin	4%
21	RiPP-like	109,850-120,065	informatipeptin	42%
22	Terpene	146,464-167,495	versipelostatin	5%
23	NI-siderophore	59,965-71,782		
24	Terpene	36,007-56,972		
25	melanin	2,453-13,043	melanin	40%
26	NI-siderophore	88,963-100,732	desferrioxamin B/desferrioxamine E	83%
27	NAPAA	1-20,361	ε-Poly-L-lysine	100%
28	T2PKS,T3PKS	12,219-92,280	spore pigment	83%
29	Phosphonate	11,857-24,778		
30	Lanthipeptide-class-i	1-18,358		

Table S8. Biosynthesis gene clusters of secondary metabolites from strain 549 detected by anti-SMASH.

	Type	Location	Most similar known cluster	Similarity
1	T2PKS, T1PKS	1-71,229	hedamycin	84%
2	terpene	200,479-222,352	hopene	30%
3	lanthipeptide-class-i	121,766-158,585	legonaridin	16%
4	T1PKS, RiPP-like	24,364-71,770	streptozotocin	7%
5	ectoine	154,001-164,405	ectoine	100%
6	NRPS,T1PKS	7,170-58,130	oryzanaphthopyran A	17%
7	Terpene	133,211-156,613	isorenieratene	87%
8	T1PKS	77,053-121,303	deoxyhangtaimycin	21%
9	lanthipeptide-class-iii	132,016-154,760	AmfS	100%
10	RiPP-like	116,058-127,446	streptamidine	66%
11	Terpene	40,758-63,157	geosmin	100%
12	NI-siderophore	219-8,160	desferrioxamine E	100%
13	NI-siderophore	62,812-76,650	peucechelin	20%
14	NRPS-like	49,179-90,114	glycopeptidolipid	13%
15	Melanin	29,228-39,704	melanin	28%
16	Terpene	7,154-28,311	s56-p1	3%
17	lassopeptide	1-16,668	lagmysin	100%
18	NRPS-like	1-27,149	A54145	5%
19	NI-siderophore	7,613-22,276		
20	T1PKS	1-9,079		

Figure S1. Neighbor-joining phylogenetic tree based on 16S rRNA gene sequences of strain LD-9 (1509 bp) and related type species of family *Microbacteriaceae*. Numbers at nodes indicate the level of bootstrap support (> 50%) based on 1000 replications. Bar, 2 nt substitutions per 100 nt.

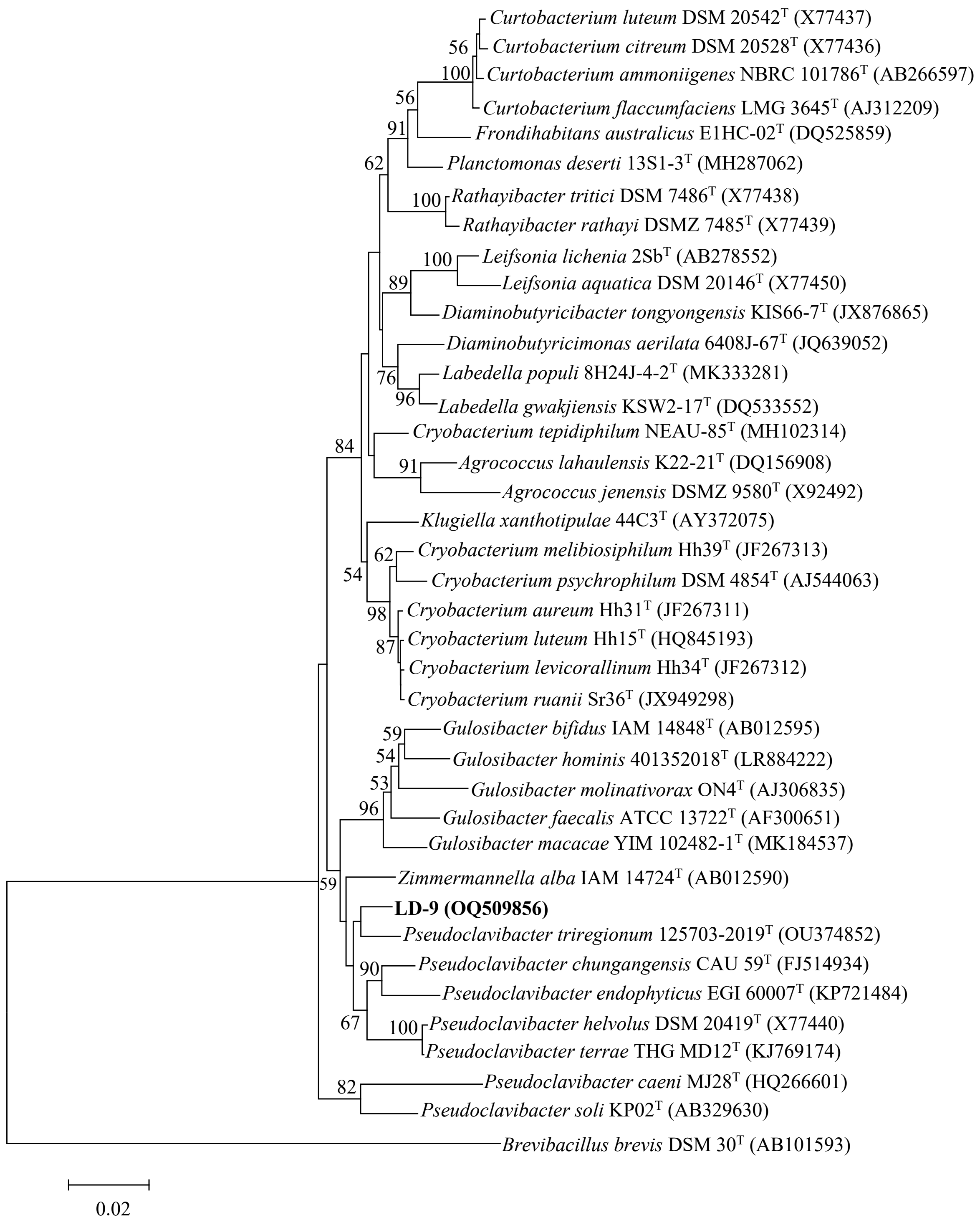

Figure S2. UPLC-MS/MS spectra of putative cosmomycin compounds (**7**-**15**) from strain 378 acquired by MS^E method. (**a**) TIC of the ethyl acetate extract of strain 378. (**b**) MS/MS spectra of nine putative cosmomycin compounds.

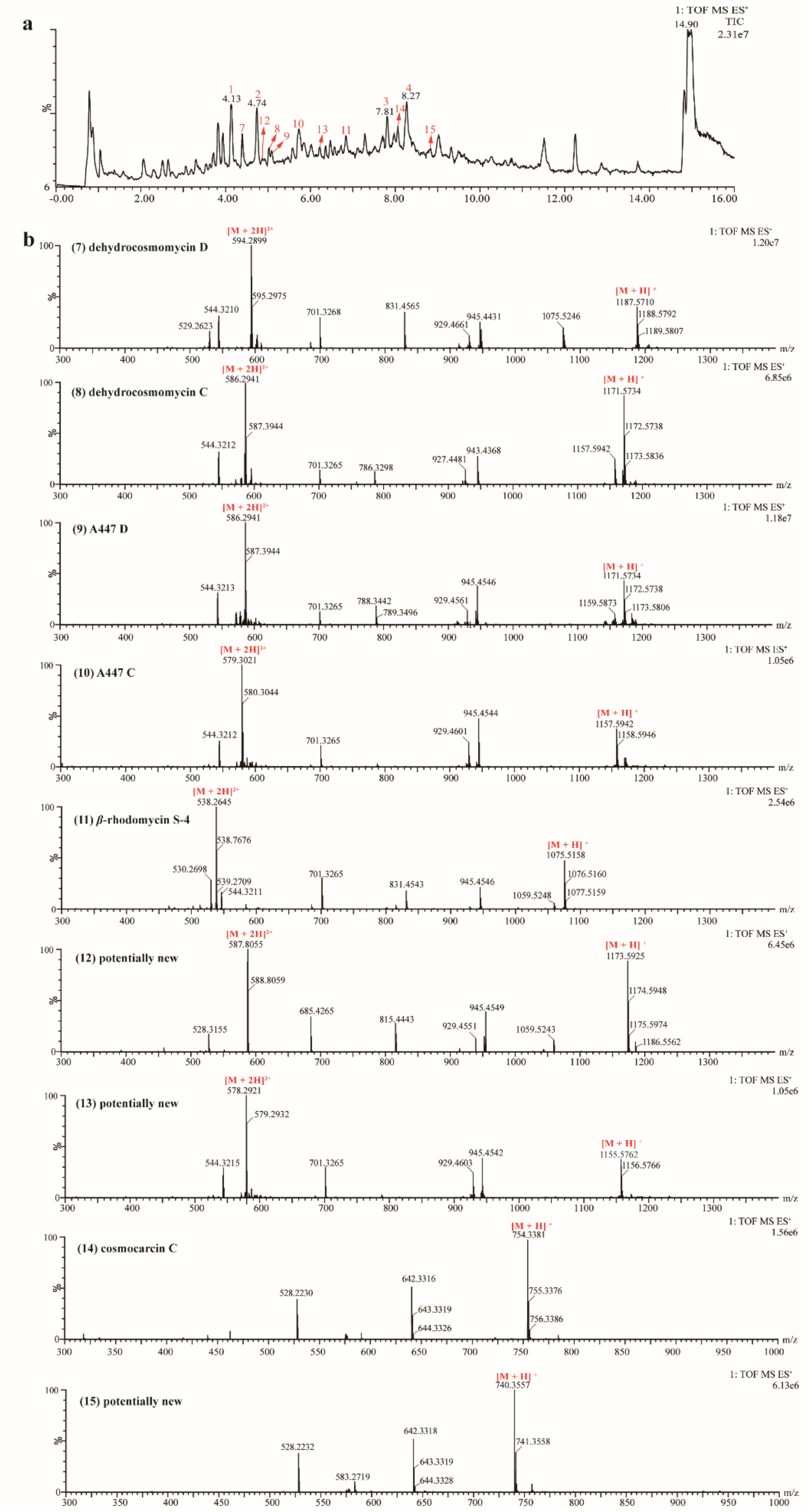

Figure S3. UPLC-MS/MS spectra of putative kidamycin derivatives (**16-21**) from strain 549 acquired by MS^E method. (**a**) TIC of the ethyl acetate extract of strain 549. (**b**) MS/MS spectra of six kidamycin derivatives.

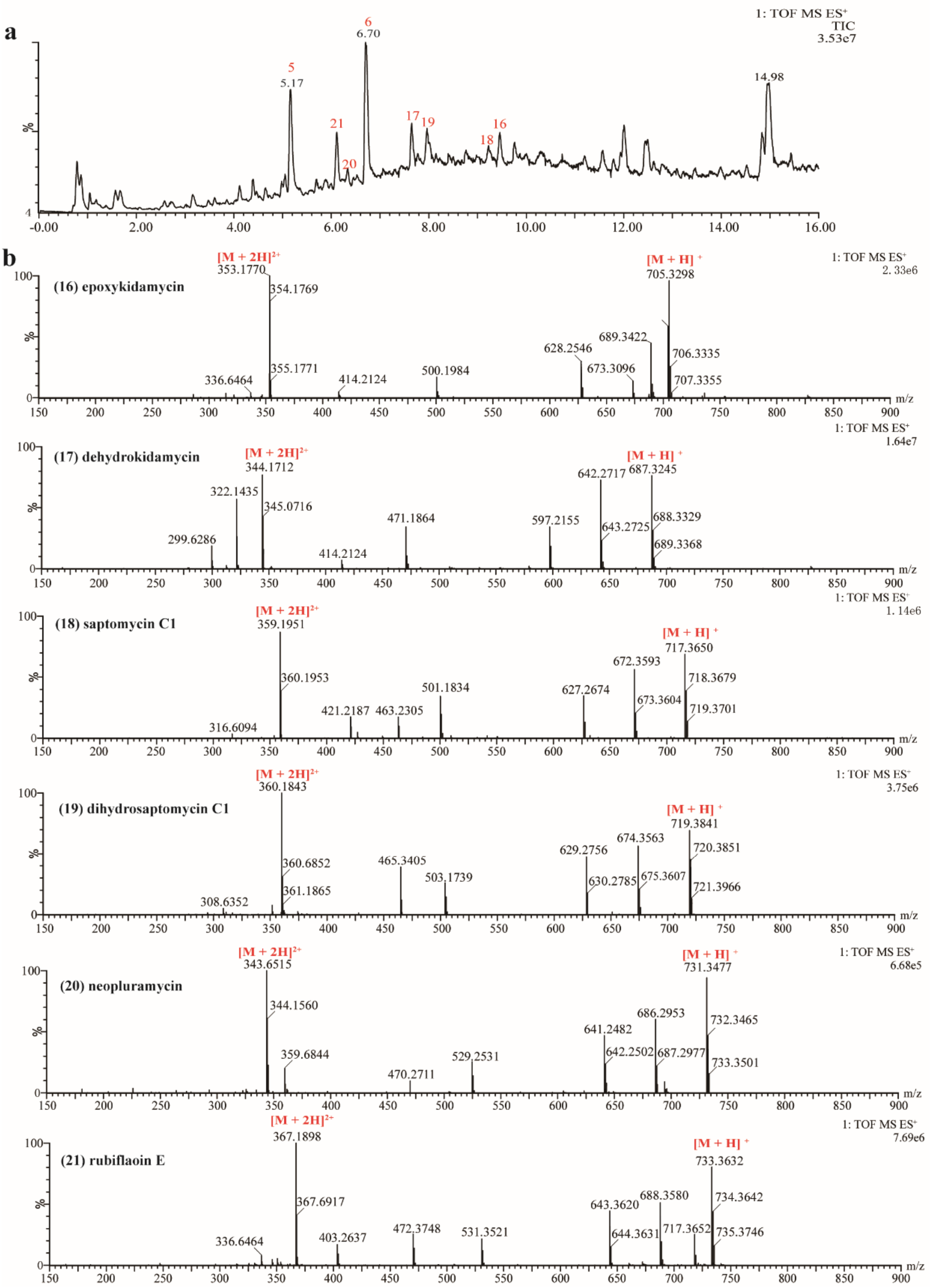

Figure S4. Similar known clusters for the putative type II polyketide BGC from strain 378.

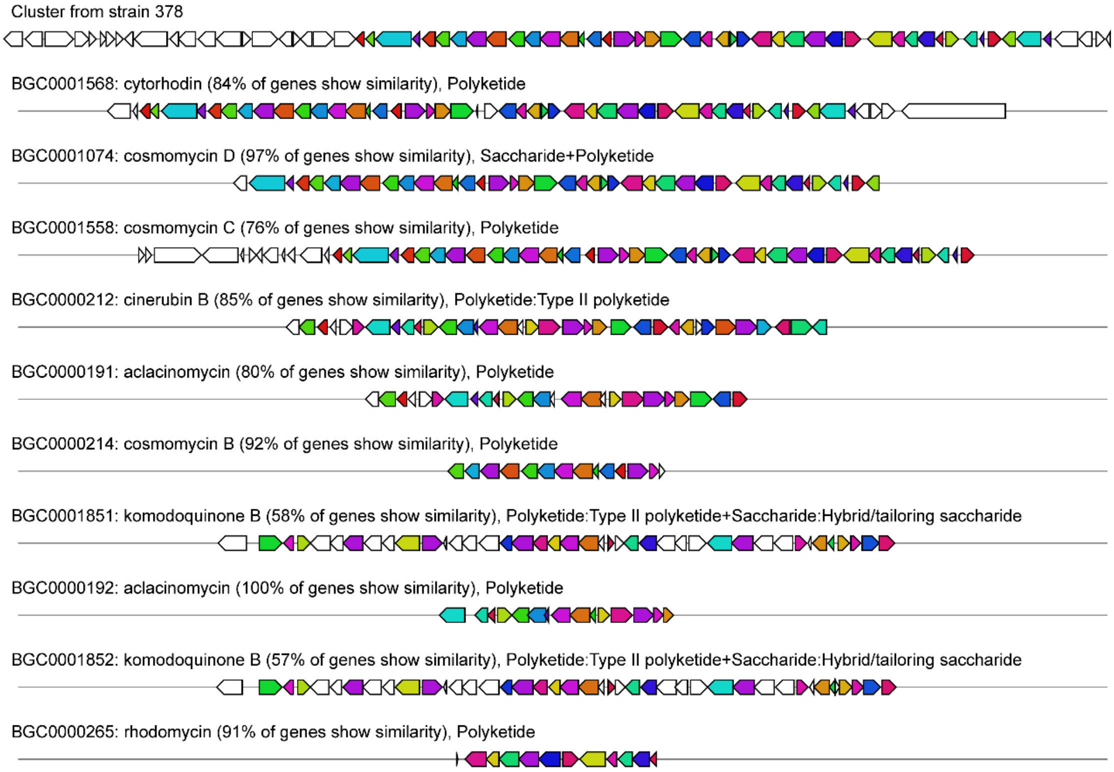

Figure S5. Similar known clusters for the putative type II polyketide BGC from strain 549.

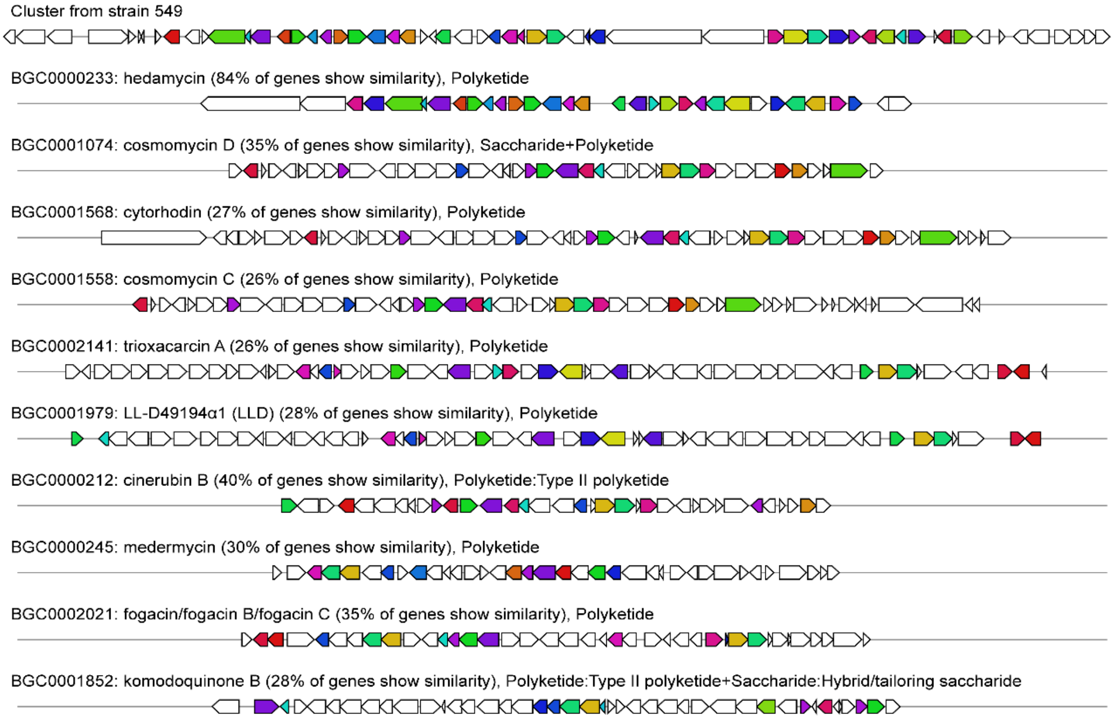

Supplement: Supplementary file 1 [file microorganisms-11-02475-s001.zip › microorganisms-2608626-supplementary.pdf]
